# 70 Years of observational weather data show increasing fire danger for boreal Europe and reveal bias of ERA5 reanalysed data

**DOI:** 10.1038/s41598-025-04200-3

**Published:** 2025-06-20

**Authors:** Johan Sjöström, Frida Vermina Plathner, Anders Granström

**Affiliations:** 1 Department of Fire and Safety, RISE Research Institutes of Sweden, Box 857, 501 15 Borås, Sweden; 2 Department of Forest Ecology and Management, Swedish University of Agricultural Sciences, 901 83 Umeå, Sweden

**Keywords:** Natural hazards, Climate and Earth system modelling, Fire ecology

## Abstract

Retrospective analyses of fire danger typically use reanalysed data, but its relation to observed fire danger is not well researched. Here we use daily weather observations to calculate fire danger for nine weather stations in Sweden, spanning 1100 km N–S, for the period 1951–2020, making it among the longest series of observed fire danger to date. All sites except the northernmost one exhibited increasing seasonal FWI-metrics over the period and linear trends were statistically significant for three sites. On average, annual peak 7-day moving-average FWI increased by 18% over 70 years. Increasing trends were mostly driven by higher noon-temperature and not by altered precipitation patterns. Further, observed fire danger differed substantially from that based on ERA5 reanalysis data. For FWI > 5, reanalysis FWI-values were on average 25% lower than corresponding observational values. The strength of reanalysis data is to form gridded data using single assimilation schemes against homogeneous model fits and it is not designed to fully represent actual point scale weather. While reanalysis data enables comprehensive geographical analyses, this study shows how it also underestimates peak fire weather in northern Europe. We recommend checking extreme-value bias against point observations in future studies.

**Supplementary Information:**

The online version contains supplementary material available at 10.1038/s41598-025-04200-3.

## Introduction

Extreme wildfire seasons and trends in wildfire occurrence are frequently attributed to climate change^1–3^. Fire danger describes the weather component relevant to fire occurrence and intensity, and the most commonly used formulation of fire danger is the Canadian Forest Fire Weather Index System (CFFWIS)^4^, often denoted as the FWI-system. Studies have shown substantial increases in occurrence of the maximum FWI-values for different regions during recent decades^5–9^. Several datasets give historical FWI on a global scale, mostly from 1979 and onwards^10–13^. These are all based on meteorological reanalysis data in which large sets of atmospheric observations are assimilated such that the interpolation scheme over all cells is homogeneous. Climate models are fitted to this assimilated set of observations to produce gridded weather data with continuous distributions in time and space. These datasets constitute powerful tools to assess historical trends and their spatial variation, and also to couple fire weather to larger meteorological phenomena^13^.

Historical reanalysed datasets are also used to benchmark projections for future decades. Global forecasts show large-scale geographical variations, mostly towards increasing fire danger^14^, with the Mediterranean and the Amazon expected to encounter the largest increases^11^. Higher precision and resolution of reanalysed data can be obtained for smaller regions such as the European territory^15,16^ or even for individual countries, e.g. Sweden^17,18^.

However, reanalysis data for any point location is a local value of a model applied to a large region, not a representation of the actual weather at point scale. There are limitations to the accuracy of historical reanalysis data as a representation of actual local weather, and biases are therefore expected^19^. The limitations depend on grid resolution and grow significantly with decreasing density of weather station data on which to construct the model^20^. Sparser observation networks lead to decreased covariance, which forces interpolated values to be over-smoothed, particularly so for extreme values. Further, when hourly data is used as input to calculate new metrics that are highly dependent on the extreme values, the mismatch between local observations and reanalysis can grow even further^21–23^. For forest fire danger, Field^24^ exemplified this mismatch by comparing fire danger based on the relatively coarse MERRA-2 reanalysis product (grid size approx. 40 × 56 km for the boreal)^25^ to fire danger based on weather observations for different regions around the world. It showed a negative mean FWI-bias for MERRA-2 compared to observations of -3.0 for North American boreal and − 1.3 for Asian boreal.

The ERA5 atmospheric reanalysis dataset^26^ (grid size approx. 9 × 9 km), widely used for detailed global analyses, also exhibits biases when compared to local weather station data. As part of a global analysis of fire weather, McElhinny et al.^27^ tested ERA5-bias using Canadian weather station data. They found a mean bias error averaging at -3.7 index-points, which skewed the data. The bias was unevenly distributed over Canada, but they did not examine further which fire danger conditions resulted in the largest bias^27^. Similarly, Phoo et al.^28^ tested the FWI-values calculated using ERA5 against data from a number of weather stations in Southeast Asia and claimed the agreement was acceptable, with overall correlations between 0.74 and 0.99 for five different weather stations.

As the phenomenon of Arctic amplification speeds up climate change at high latitudes^29^, the fire danger in the north has been suggested to increase faster than elsewhere^30^, but for boreal Europe this has not yet been shown. The climate changes observed so far include increasing temperatures in all seasons, but strongest in the winter, as well as increasing precipitation primarily during the winter and summer (JJA)^31,32^. The number of days with snow cover has decreased significantly in the southern part of boreal Europe^33^. Further, these changes have occurred mainly over the last three decades but so far, the net effect of these changes on fire danger has not been analysed.

In contrast to most of the circumboreal region, Sweden has a dense network of high-quality weather stations, many of them operational since 1860^34^. However, FWI-index calculations require 10-m open wind speed and relative humidity (or dew point temperature from which to calculate RH), in additions to daily temperature and precipitation records. These quantities were recorded only from the early 1950s. Here we utilise daily weather observations from nine weather stations spanning a N–S gradient of 1100 km throughout Sweden to produce a 70-year-long time series of the FWI index, delineating multi-decadal trends of actual observed fire danger in Sweden. These stations have exceptionally good historical continuity, with only minor hiatuses and few changes of locations or observation techniques. They have continuous records of noon-temperature and daily precipitation which we utilise to produce drought-centered fire-danger metrics stretching back to 1860, to be reported elsewhere.

In this paper we use the full set of weather observations required for assessing FWI to produce continuous series of fire danger, covering 70 years of actual observations for these sites, which we then compare to ERA5-based calculations of fire danger. Our research objectives are to (1) identify long-term trends in observational fire danger for these stations and assess which subindices and moisture codes they might stem from and (2) quantify systematic differences between fire danger calculated from actual observations and that calculated from gridded reanalysis data.

## Method

### The CFFWIS structure

The Canadian Forest Fire Weather Index (FWI) System is a widely used tool for assessing forest fire danger. First, it evaluates the moisture content of three fuel layers based on daily (noon) weather conditions^4^. Each fuel layer is described by a moisture code that can be translated to actual moisture contents. The fine fuel moisture code (FFMC) describes the moisture content of litter and fine fuels (approximately 250 g/m^2^). It is driven by temperature, relative humidity, wind speed, and precipitation. The duff moisture code (DMC) use temperature, relative humidity, and precipitation to assesses the average moisture content of a loosely compacted sub-surface organic layer (~ 5 kg m^−2^). The drought code (DC) models moisture content of deep, compact organic layers (~ 25 kg m^−2^). Entry variables for calculating DC are only temperature and precipitation, and high code values indicate seasonal drought^4^. Response times differ markedly for the three indices. Under “standard” summer conditions without precipitation 2/3 of the free moisture is lost in 1.5 days for FFMC, in 15 days for DMC and in 53 days for DC^4^.

By combining FFMC and current wind speed, the initial spread index (ISI) estimates the fire rate of spread. A secondary moisture index, the buildup index (BUI), estimates the total amount of fuel available for combustion, by combining the DMC with a fraction of the DC code value. Finally, the fire weather index (FWI) combines ISI and BUI to provide an index related to the fire-line intensity.

### Weather data

**Fig. 1:**
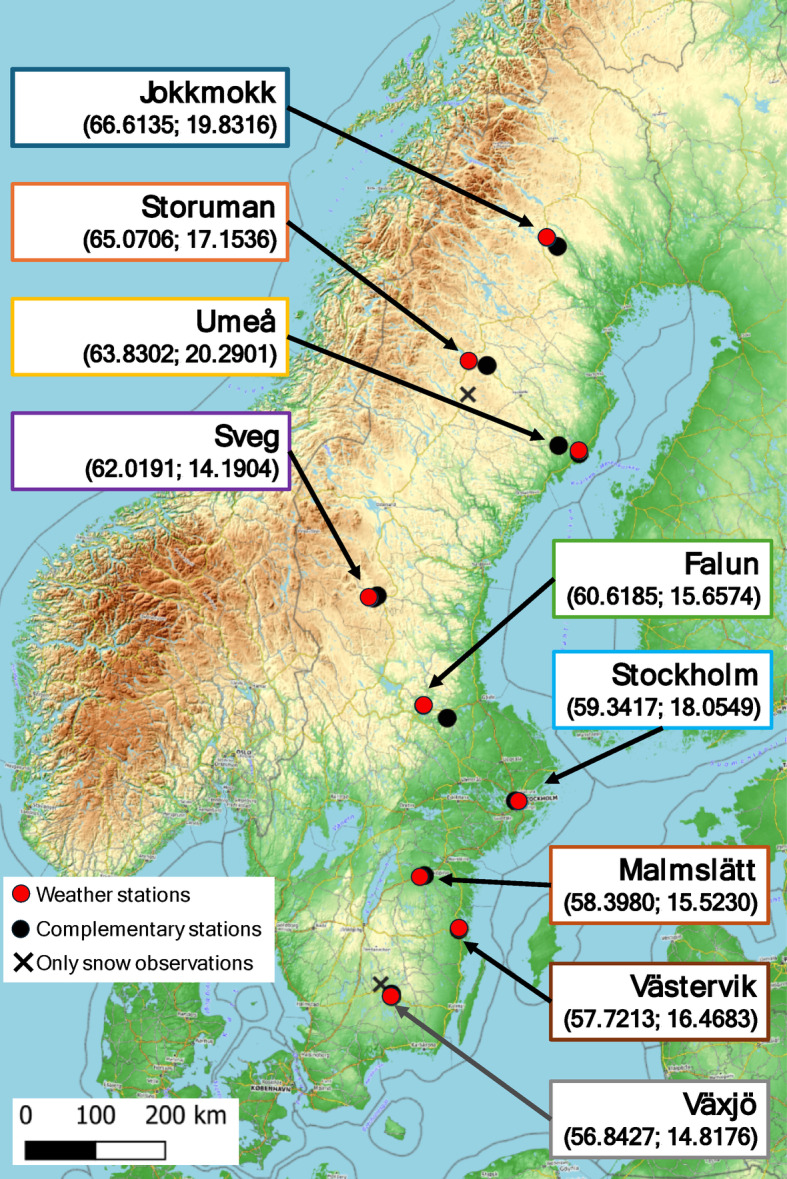
Site locations. Topographic map of Scandinavia with the locations of the weather stations (red dots), complementary stations (black dots) and complementary stations only used for snow cover (crosses). Coordinates of the main stations (WGS84) are detailed in the boxes. The map was created using QGIS 3.34.0-Prizren (https://qgis.org/) with OpenTopography map(https://opentopography.org/) from OpenStreetMap, with permission (CC BY-SA 2.0).

We collected daily observations from nine weather stations in Sweden spanning the period from 1951 to 2020. These stations register noon (12.00 UTC) observations of 2-m air temperature, 2-m relative humidity (RH) (or 2-m dew point temperature, which we translated to RH as detailed by Magnus^35^) and 10-m open wind speed (averaged over previous 10 min). The stations also provided 18.00 UTC observations of past 24-h cumulative precipitation (standard in the Swedish system) as well as depth and state of snow cover, which determine when calculations should start^36^. The stations span a 1500 km N–S distance from Jokkmokk in the north to Växjö in the south (Fig. 1). Further, as part of the attribution of fire danger changes to weather, we calculated the partial vapour pressure of water, vp_w_ at noon, based on temperature and relative humidity, using equations provided by the International Association for the Properties of Water and Steam.

If data was missing at 12.00 UTC we used the closest available observation at the same station of maximum 6 h difference. If this was also missing, the 12.00 observation from the closest weather station was used after bias-correction using correlations between the original and closest station for data when overlap occurred. Data was to > 99% reported for all variables at the correct hour, reducing any possible error from the complementary stations.

### Fire danger calculations

For each site and year, the calculation of fire danger was initiated three days after the last snow period^37^ which here is defined as at least five days of snow cover. Snow cover is, in turn, defined by a snow depth of ≥ 3 cm on at least 50% of the ground at the observation point. For snow-free winters in the south, we instead initiated the model runs March 1st. Earlier research^38,39^ has established that there is negligible fire danger in the period October-February even if there is no snow, because of high humidity and low rate of evaporation. Preliminary testing revealed that the specific choice of model start date had no significant effect on the summer fire danger result, as shown also by Berg et al^18^. We stopped running the model at September 30th each season. The full set of indices of CFFWIS were calculated according to Van Wagner and Pickett^40^ without the Drought Code (DC) overwintering component^4^, using Matlab.

7-days maximum of FWI was chosen to characterize each year’s fire season. Hereafter, the peak value of the 7-day moving average is denoted using suffix “-7x” (e.g. FWI-7x). The use of FWI-7x as a seasonal characteristic follow recent international literature on the effect of climate change on fire danger^1,41^. In fact, FWI-7x correlates, for our data set, to the Seasonal Severy Rating (SSR, from Van Wagner and Pickett^40^) with a Pearson correlation coefficient of 0.87 but we chose FWI-7x as a more straightforward index of peak fire danger. Trends in fire danger and weather over the full 70-year period were determined using linear fits as we could not find other temporal patterns across the different sites.

As for the wind peak (W-7x), we only assessed this for days when we assume, based on index levels, that surface fuels were dry-enough to carry fire. For this we set a threshold at Fine Fuel Moisture Code (FFMC) > 85 (representing the moisture of extinction for fire in boreal surface fuels) and a Duff Moisture Code (DMC) > 20.

We also analysed if there were seasonal shifts in peak fire danger (i.e. timing of maximum 7-days average per season). Therefore, FWI-7x assessed over each month were also calculated and the linear 70-years trends of FWI-7x were thus assessed for each site and within each month (May–September) separately.

### Contribution of subindices, moisture codes and weather parameters to long-term changes in fire danger

The seasonal peaks of the weather parameters or moisture codes are not necessarily concurrent with the peak FWI^18^. To find the underlying contributions to changes in FWI-7x, we assessed the values of the subindices and moisture codes concurrent with the period of FWI-7x for each season. We used this more ad-hoc approach to examine the background of changes in fire danger as our data contains only nine point locations and statistical assessments, such as the Mann–Kendall test, therefore are of limited value. Thus, we extracted the 7-day averages of these subindices and codes from the same seven days during which the FWI-7x occurred, as the FWI value is directly related to the concurrent values of subindices and codes, here labelled ISI-7_m_, BUI-7_m_, FFMC-7_m_, DMC-7_m_, DC-7_m_, where subscript “m” refers to the period of maximum FWI. The trend over the 70-year period 1951–2020 for the annual values of subindices and codes during peak seasonal FWI was then approximated by linear regressions, the trend labelled ${fwi}_{y}$, ${isi}_{y}$, ${bui}_{y}$, ${ffmc}_{y}$, ${dmc}_{y}$, and ${dc}_{y}$, for year $y$ (variables and fits are found in supplementary information section 4). We used a multivariate linear regression (Matlab ‘fitlm’ algorithm) to model the relation between FWI-7x and these concurrent variables. The linear trend of FWI-7x (${fwi}_{y}$) was separated into contributions from ${isi}_{y}$ and ${bui}_{y}$ according to the following steps:

1. Fit a linear regression of FWI-7x to the predictor variables ISI-7_m_ and BUI-7_m_.

1 $${\text{LM}}_{FWI}={\beta }_{0}+{\beta }_{1}{\text{ISI-7}}_{m}+{\beta }_{2}{\text{BUI-7}}_{m}$$

(2) Attribute the changes in FWI-7x from ISI and BUI using the linear trends in subindices

2 $$\begin{aligned} & \Delta_{isi}^{fwi} = \beta_{1} \left( {isi_{2020} - isi_{1951} } \right) \\ & \Delta_{bui}^{fwi} = \beta_{2} \left( {bui_{2020} - bui_{1951} } \right) \\ & \Delta_{res}^{fwi} = fwi_{2020} - fwi_{1951} - \Delta_{isi}^{fwi} - \Delta_{bui}^{fwi} \\ \end{aligned}$$

where ${\Delta }_{res}^{fwi}$ describes the residual of ${fwi}_{y}$ not captured by the linear trends of ${isi}_{y}$ and ${bui}_{y}$.

Thereafter, using the same algorithms, we calculated how the linear change in ISI was attributed to trends in FFMC, wind and residuals (${\Delta }_{ffmc}^{isi}$, ${\Delta }_{w}^{isi}$ and ${\Delta }_{res}^{isi}$) as well as the corresponding attribution for BUI (${\Delta }_{DMC}^{bui}$, ${\Delta }_{DC}^{bui}$ and ${\Delta }_{res}^{bui}$). Combining these contributions, we were able to derive the contribution of trends in moisture codes and wind to the trends in FWI-7x. The attributions of the linear change in FWI-7x to trends in subindices and moisture codes were done regardless of whether the linear approximations were statistically significant or not.

Attributing the trends in moisture codes to trends in the different weather parameters is more complicated since the code values are not directly related to concurrent weather parameters but are subject to different time lags. Additionally, temperature and relative humidity are highly correlated, making attribution to one or the other difficult. We therefore derived estimates of weather contribution to ${ffmc}_{y}$ from concurrent temperature (${t}_{y}$), water vapour pressure (${vp}_{wy}$) and wind (${w}_{y}$) using the same type of regression as for the subindices and moisture codes. Temperature and water vapour pressure have a low degree of correlation, and we assume the time since rain for peak FWI periods to be much longer than typical lag-times of FFMC (i.e. 1.5 days). As above, the difference between these model changes (${\Delta }_{t}^{ffmc}$, ${\Delta }_{vp}^{ffmc}$ and ${\Delta }_{w}^{ffmc}$) and the linear FFMC-trend (${ffmc}_{y}$) was attributed to a residual contribution (${\Delta }_{res}^{ffmc}$).

Similarly, the influence of T, vp_w_ and precipitation on DMC was estimated through linear regression against concurrent T, vp_w_ and ${P}_{DMC}\left(y\right)$, the annual precipitation ‘absorbed’ by the DMC code until peak fire danger. We calculated ${P}_{DMC}\left(y\right)$ by the annual cumulative precipitation until the day of fire danger maximum, weighted by an exponential decay of 15 days and a threshold of 1.5 mm/24-h, as daily precipitation < 1.5 mm does not influence the DMC.

3 $${P}_{DMC}\left(y\right)={\sum }_{d={d}_{start}\left(y\right)}^{{d}_{max}\left(y\right)}\text{exp}\left(\left(d-{d}_{max}\left(y\right)\right)/15\right)\times \left(P\left(d\right)-1.5\right)$$

where ${d}_{start}\left(y\right)$ and ${d}_{max}\left(y\right)$ are the day-of-year for the start of fire danger calculations and the maximum of FWI-7 for year $y$, respectively and $P\left(d\right)$ is the daily precipitation of day $d$. Note that $P\left(d\right)-1.5$ had a lower cap of 0.

Finally, trends in DC were attributed to trends in temperature and precipitation by linear regressions against the average noon temperature of the season until the day of maximum FWI-7 as well as the cumulative precipitation until the same day, weighted by a 53-day exponential decay and a precipitation threshold of 2.8 mm/24-h (the minimum daily precipitation that affects DC). The estimations of weather parameter’s influence on the moisture codes were combined with the influence of moisture codes to FWI, indicating how weather parameters influenced potential changes in FWI.

### Fire danger calculations using observation versus re-analysed data

For comparison with the observation data, 12.00 UTC weather parameters and 18.00 UTC past 24-h precipitation were extracted from the ERA5 atmospheric reanalysis dataset for the cells overlapping the weather station locations^26^. Fire danger was calculated using these weather inputs, but with the same starting dates as for the observational data. The same seasonal extremes were defined, and the trend evaluated using linear fits to the 70-years period. Observation- and ERA5-based FWI-values for each day and site were compared and correlated to assess systematic differences between the two datasets.

## Results

### Weather observations

The disappearance of the snowpack showed only small variations for the northern sites, with model start day in early May for the northernmost station (Jokkmokk). For stations south of latitude 60°, variation between years increased and onwards from 1989 there was a clear shift, with 10–17 days earlier snow melt compared to the earlier period. See Supplementary information (Supplementary Figure S1) for details on this time series.

For the weather trends, the sites show both commonalities and differences, not always with clear geographical trends (Table 1). Linear regressions show increasing T-7x (max 7-days average of noon-temperature) for all sites, with an average increase of 1.6 °C over the 70-year period, but with no apparent N–S difference. Four of these trends were statistically significant at a significance level of 0.05, and were as large as + 2 to + 3 °C in the southeast (Stockholm, Malmslätt and Västervik) but also at the northern site of Storuman.

**Table 1:** Changes evaluated by linear regression over the period 1951–2020 for each seasonal 7-days maximum of weather indicators and precipitation related metrics.

Site	Veg. period (days)^a^	T-7x (°C)	RH-7x (%)	W-7x (ms^−1^)^b^	Tot prec. (JJA) (mm)	Longest drought (JJA) (days)^c^
		Change from linear regression 1951–2020				
Jokkmokk	135	+ 0.1	+ 1.6	− 0.5	+ 56*	+ 1.2
Storuman	130	+ 2.6*	− 11*	− 0.4	+ 6.3	+ 0.3
Umeå	165	+ 0.7	− 3.0*	+ 0.3	− 19	− 1.0
Sveg	160	+ 1.5	− 3.6	− 1.0*	+ 12	− 1.2
Falun	180	+ 0.5	− 11*	+ 0.7*	− 5.6	+ 1.9
Stockholm	205	+ 3.0*	− 3.7	− 1.4*	− 1.5	− 0.5
Malmslätt	205	+ 2.0*	− 3.2	+ 0.0	+ 45	− 3.9*
Västervik	210	+ 3.1*	− 12*	− 0.3	+ 53*	+ 0.8
Växjö	205	+ 0.7	− 4.4*	+ 0.7*	+ 32	+ 2.6
Average	175 days	+ 1.6 °C*	− 5.6%*	− 0.2 ms^−1^	+ 19.8 mm	± 0.0 days

The average vegetation period for each site refer to the period 1991–2020. Statistically significant (*p* < 0.05) linear trends are marked with (*).

^a^See Schimanke et al^32^.

^b^Only evaluated for days with FFMC > 85 and DMC > 20.

^c^Longest period of consecutive days without precipitation.

Summer precipitation (JJA) also increased for most sites, with an average of + 20 mm/season, corresponding to roughly a 10% increase over the 70-year period. The increase was largest for the northernmost site Jokkmokk as well as for the three most southern sites but only statistically significant for two sites.

All sites except for Jokkmokk exhibited a clear decreasing trend of the minimum noon relative humidity, RH-7x. The decrease averages at -5.6%-units which corresponds to 1/6 of the mean value (Table 1). Each season’s peak wind period during flammable conditions, W-7x, exhibit mostly decreasing or negligible trends over the period.

### Observation-based fire danger

**Fig. 2:**
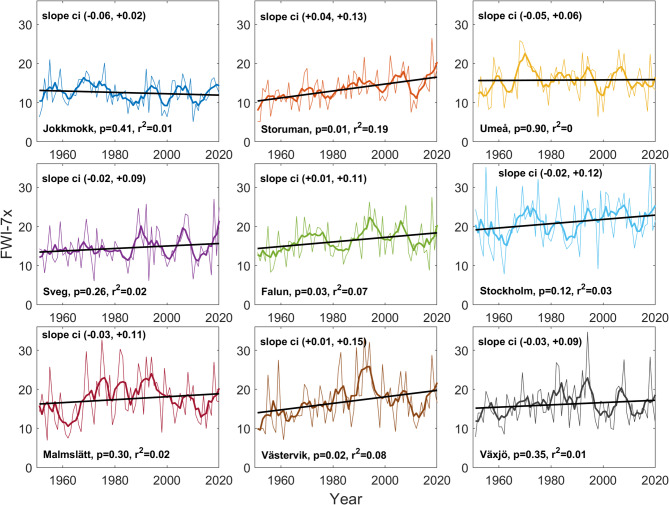
Annual values of FWI-7x for the nine sites (thin lines). Trends are shown through 5-year averages (thick lines) and linear trends (black lines).

Despite large inter-annual variation in FWI-7x (s.d. between 27 and 36% of the mean), linear trends were positive for all sites except for the northernmost one (Jokkmokk, Fig. 2), although linear trends were statistically significant (*p* < 0.05) only for three sites (Table 2). Likewise, ISI-7x, FFMC-7x, BUI-7x and DMC-7x increased for all stations except for Jokkmokk (Table 2).

**Table 2:** Linear trends over 70 years for each seasonal 7-days maximum of code values from CFFWIS, the seasonal length and severity rating (SSR). Statistically significant (*p* < 0.05) linear trends are marked with (*).

Site	FWI-7x	ISI-7x	BUI-7x	FFMC-7x	DMC-7x	DC-7x	Season length^a^	SSR^b^
	Linear change (code value) 1951—2020							
Jokkmokk	− 1.2	+ 0.19	− 12	+ 0.2	− 8.2	− 32	− 9.8	− 0.27
Storuman	+ 6.1*	+ 1.89*	+ 17*	+ 3.0*	+ 17*	+ 28	+ 17.4*	+ 0.47*
Umeå	+ 0.2	+ 0.26	+ 1.7	+ 0.0	+ 0.0	+ 32	+ 1.8	− 0.10
Sveg	+ 2.2	+ 0.78	+ 6.2	+ 1.2	+ 4.0	+ 10	− 2.2	+ 0.12
Falun	+ 4.0*	+ 1.99*	+ 0.78	+ 2.2*	+ 6.3	+ 24	+ 12.0	+ 0.36*
Stockholm	+ 3.8	+ 0.59	+ 12	+ 1.2	+ 10	+ 45	+ 12.5	+ 0.10
Malmslätt	+ 2.6	+ 0.38	+ 13	+ 0.8	+ 12	+ 8.8	+ 1.4	+ 0.03
Västervik	+ 5.7*	+ 2.01*	+ 8.0	+ 2.8*	+ 14	− 29	+ 10.2	+ 0.36
Växjö	+ 2.0	+ 0.90*	+ 13	+ 0.7	+ 13	+ 24	+ 2.1	+ 0.27
Average	+ 2.9*	+ 1.00*	+ 6.6	+ 1.3*	+ 7.5	+ 12	+ 5.1*	+ 0.15

^a^Defined by number of days with FWI > 8.

^b^The seasonal severity index for summer (JJA) according to Van Wagner and Pickett^40^.

Across all sites, the average FWI-7x increase was statistically significant, with a mean increase of 18% above the mean period value (confidence interval 0%—37%), with the largest changes at Storuman (45%) followed by Västervik (35%), Falun (25%) and Stockholm (20%). For most sites 5-year averages showed a tendency towards multi-year regimes relative the linear trend, but without any detectable periodicity (Fig. 2), see e.g. Umeå 1966–1976 or Malmslätt 2002–2017.

**Fig. 3:**
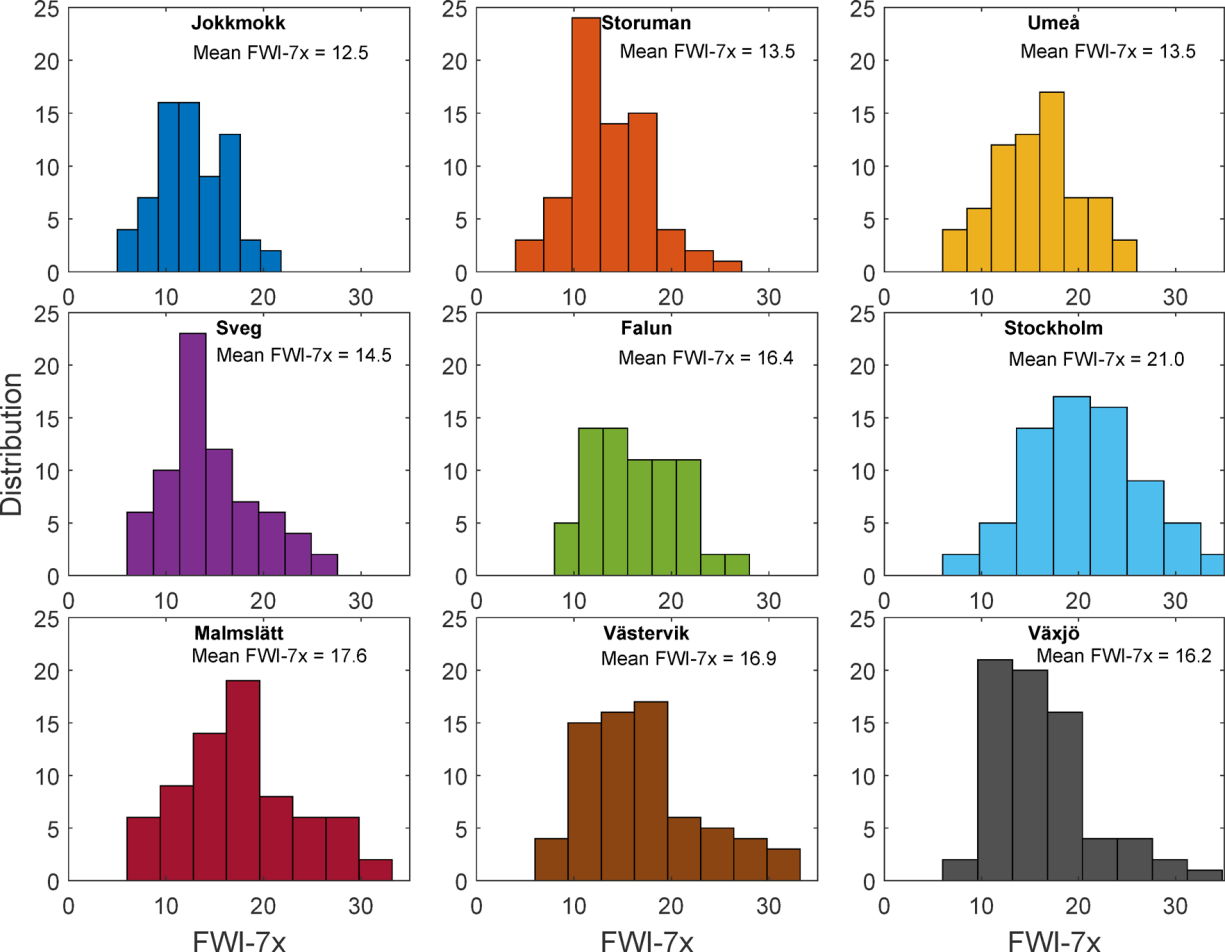
Histograms of FWI-7x for each site over the 70 studied seasons (8 bins).

**Fig. 4:**
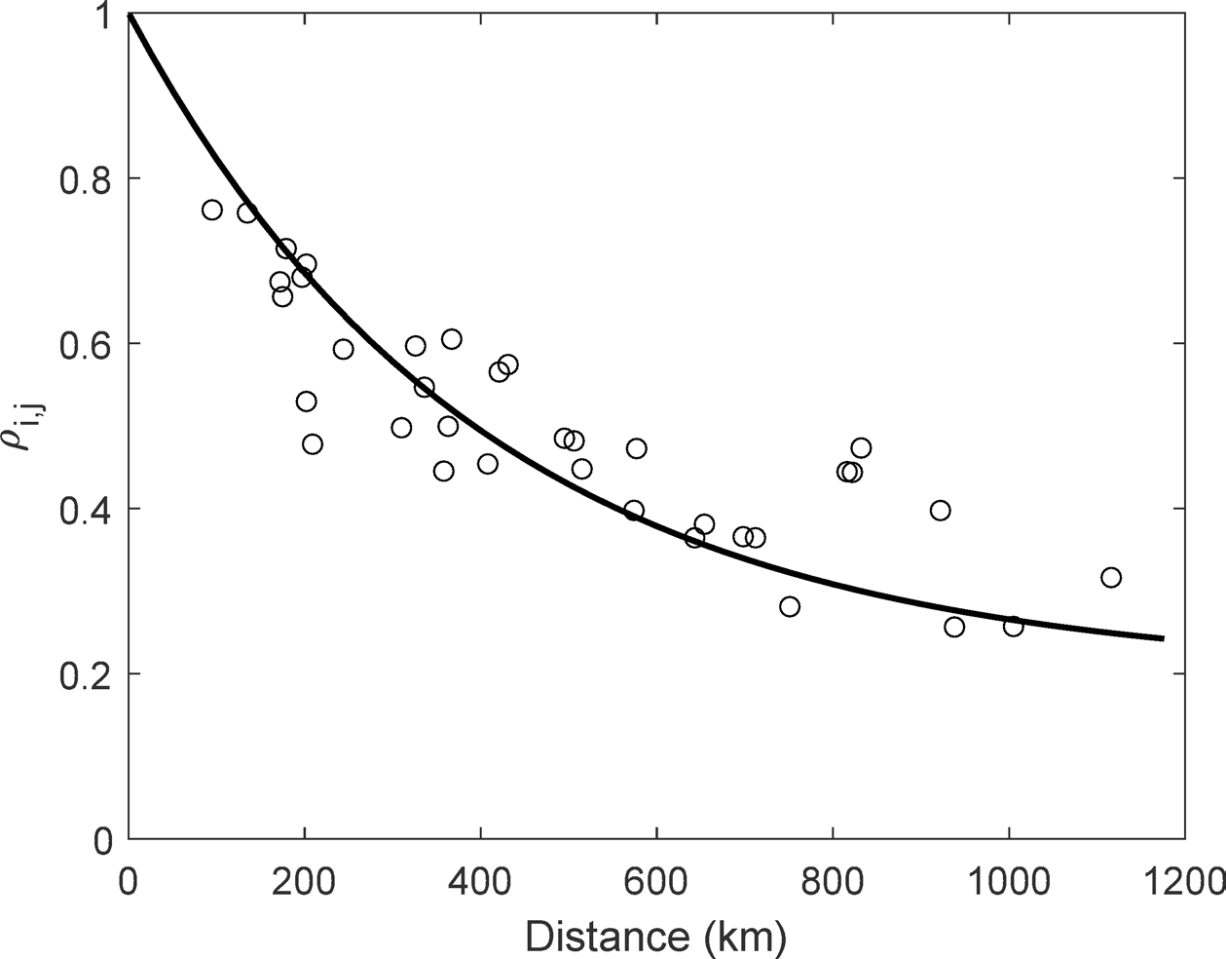
Pearson correlation between sites for annual FWI-7x plotted against distance between the sites. The line represents an exponential fit to the data: ${\rho }_{i,j}={\text{exp}}\left(-D/400\right)+0.2$

The mean of the 70-year FWI-7x distributions ranged 8.5 units between stations, from 12.5 at Jokkmokk in the inland north to 21 at Stockholm in the southeast (Fig. 3). The correlation of the yearly temporal variation in FWI-7x between the different sites, assessed through the Pearson correlation matrix, varied from 0.26 to 0.76 (Supplementary Table S3). Correlation decreased exponentially with distance between sites (Fig. 4).

### Contributions to FWI-trends

**Fig. 5:**
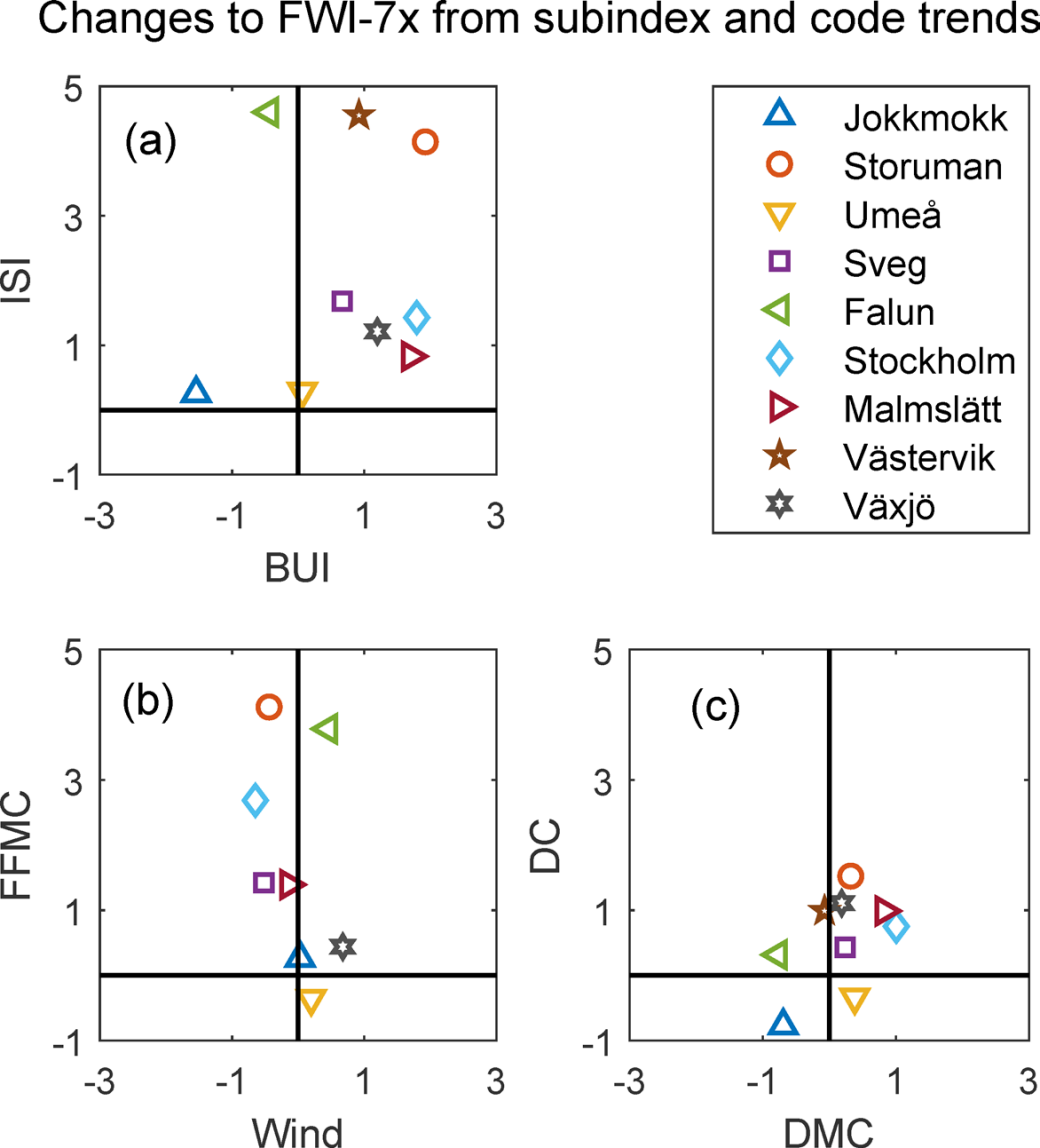
Contributions to the long-term trend in FWI-7x from linear approximations of subindices and moisture codes. (**a**) BUI and ISI. (**b**) Wind and FFMC. (**c**) DMC and DC.

For all sites, the linear regression models of FWI-7x were statistically significant to concurrent ISI and BUI. All time-series of ISI and BUI could however not be estimated by a linear function with *p* < 0.05. Concurrent increase in ISI contributed more to the increasing trend in FWI-7x than did changes in BUI, for all sites except Jokkmokk, Stockholm and Malmslätt. Only the northernmost Jokkmokk exhibited a substantial negative contribution from BUI (Fig. 5a). Most often, the FFMC-component of ISI had the largest influence on increasing FWI-7x compared to the other moisture codes (Fig. 5c) and concurrent wind. (Fig. 5b). Trends in wind had, on average, a negligible effect on trends in FWI-7x; it contributed positively for some sites and negatively for others.

**Fig. 6:**
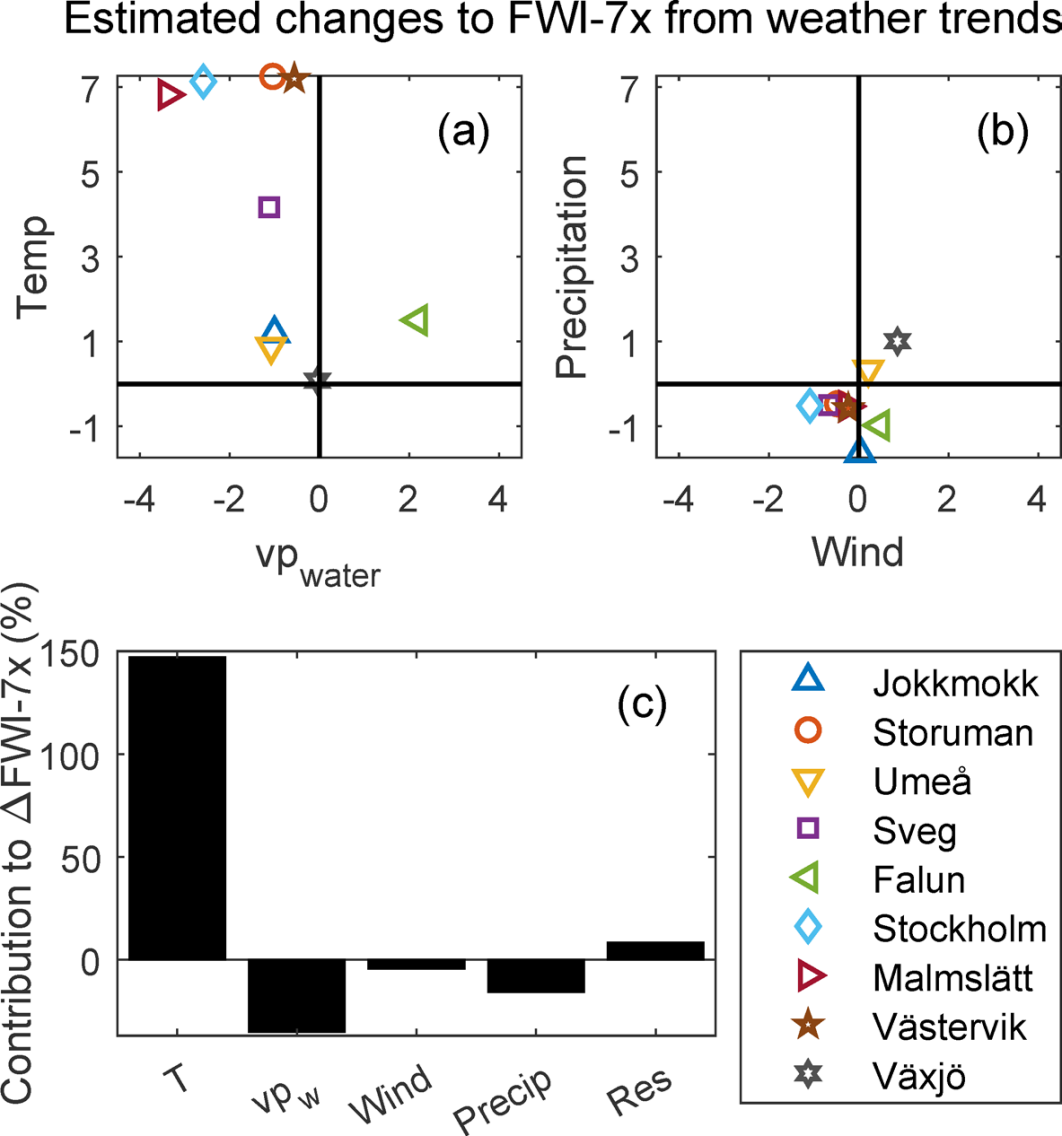
Relative contribution of individual weather parameters to the long-term linear trends in FWI-7x. (**a**) Temperature and water vapour pressure. (**b**) Precipitation and wind (**b**). Panel (**c**) shows the relative contributions from the weather parameters averaged over all sites. The category “Res” (Residual) includes changes in FWI which could not be captured by the linear model we used.

The estimates of the contribution of different weather parameters to changes in moisture codes (i.e. FFMC, DMC and DC) and in turn to the FWI-index, suggest that temperature was the most influential for the changes in FWI-7x (Fig. 6). Increasing temperatures during the peak fire danger period explained 147% of the linear trend in FWI-7x averaged over all sites. The water vapour pressure contributed negatively for all sites but one and balanced the positive influence from temperature by − 34% on average. Likewise, the average contributions from wind and precipitation were negative, − 4% and − 18%, respectively. The residual contribution, not described by the linear models, was on average less than + 9% (Fig. 6c).

### Seasonal trends

**Fig. 7:**
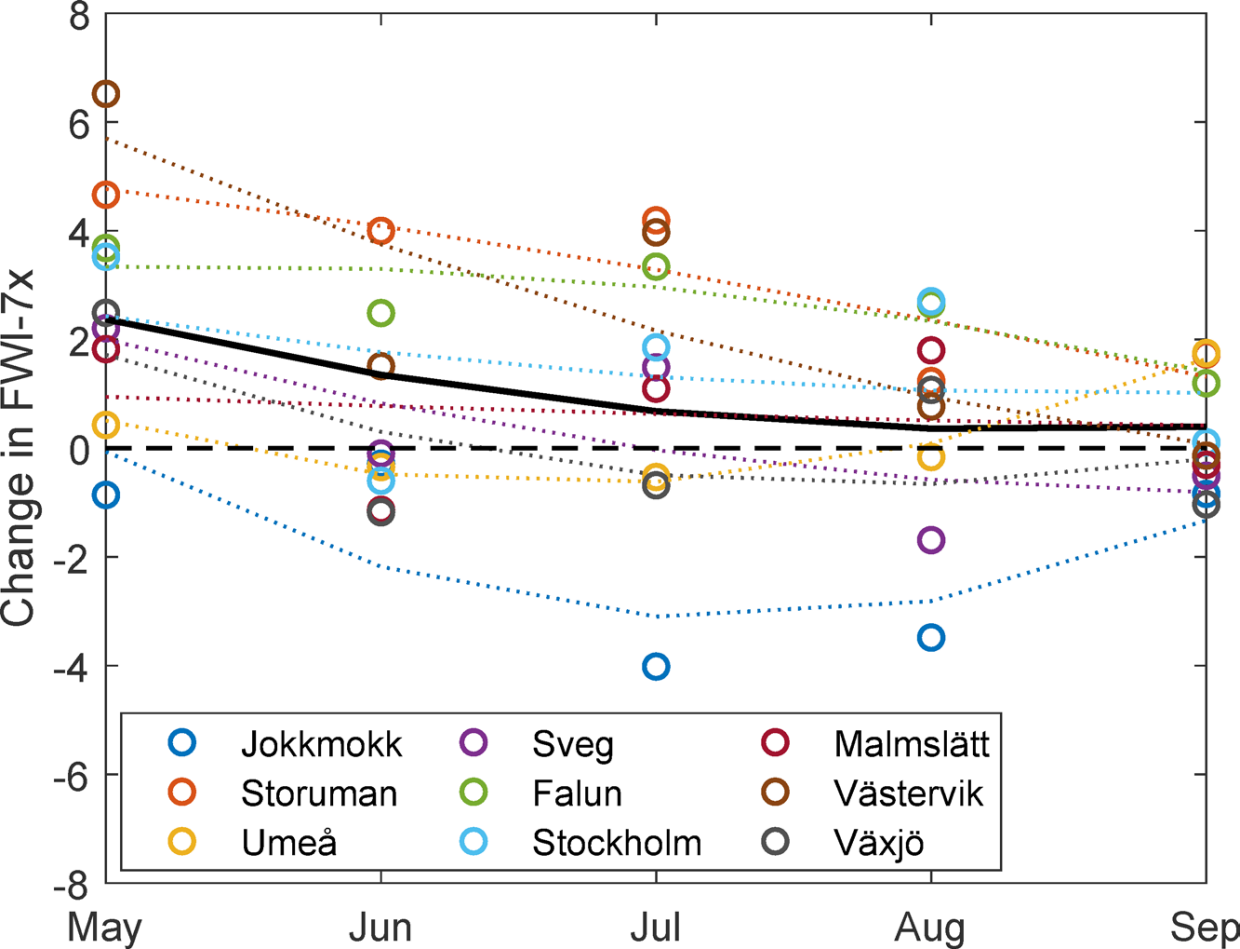
Linear change over 70 years of monthly FWI-7x for all sites. Dashed curves represent 2nd order polynomial fit for each site. The black line represents 2nd order fit to all sites combined.

**Fig. 8:**
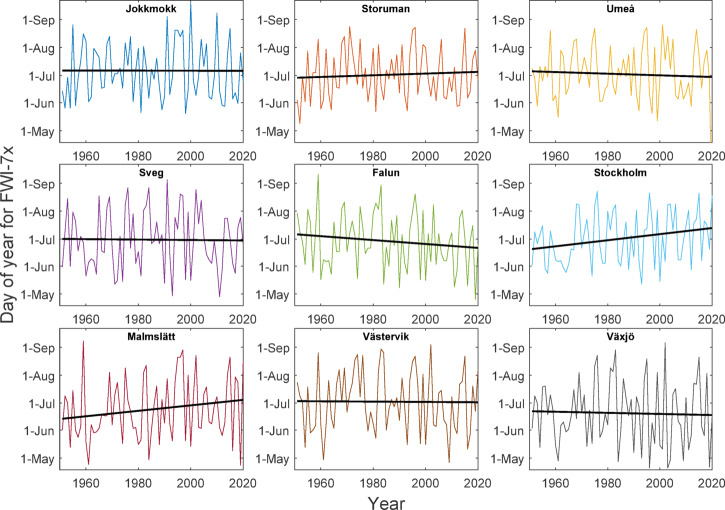
Timing of FWI culmination. The day of year during which the highest 7-day average of FWI (FWI-7x) occurred (thin lines) and linear trend (black lines).

The 70-year linear trends in FWI-7x, broken down by month for the different sites from May to September, was largest in the early season and decreased throughout the summer, when averaged over all sites (Fig. 7). The larger changes in FWI for the early season would be expected to influence the seasonal timing of FWI-7x, but for most sites there were only minor shifts of the fire danger culmination date compared to the interannual variations (Fig. 8). Two sites, Stockholm and Malmslätt, had marked trends towards later timing of FWI-7x, with a 23- and 21-day shift respectively over the 70-year period.

### Observation-based vs reanalysis-based fire danger

**Fig. 9:**
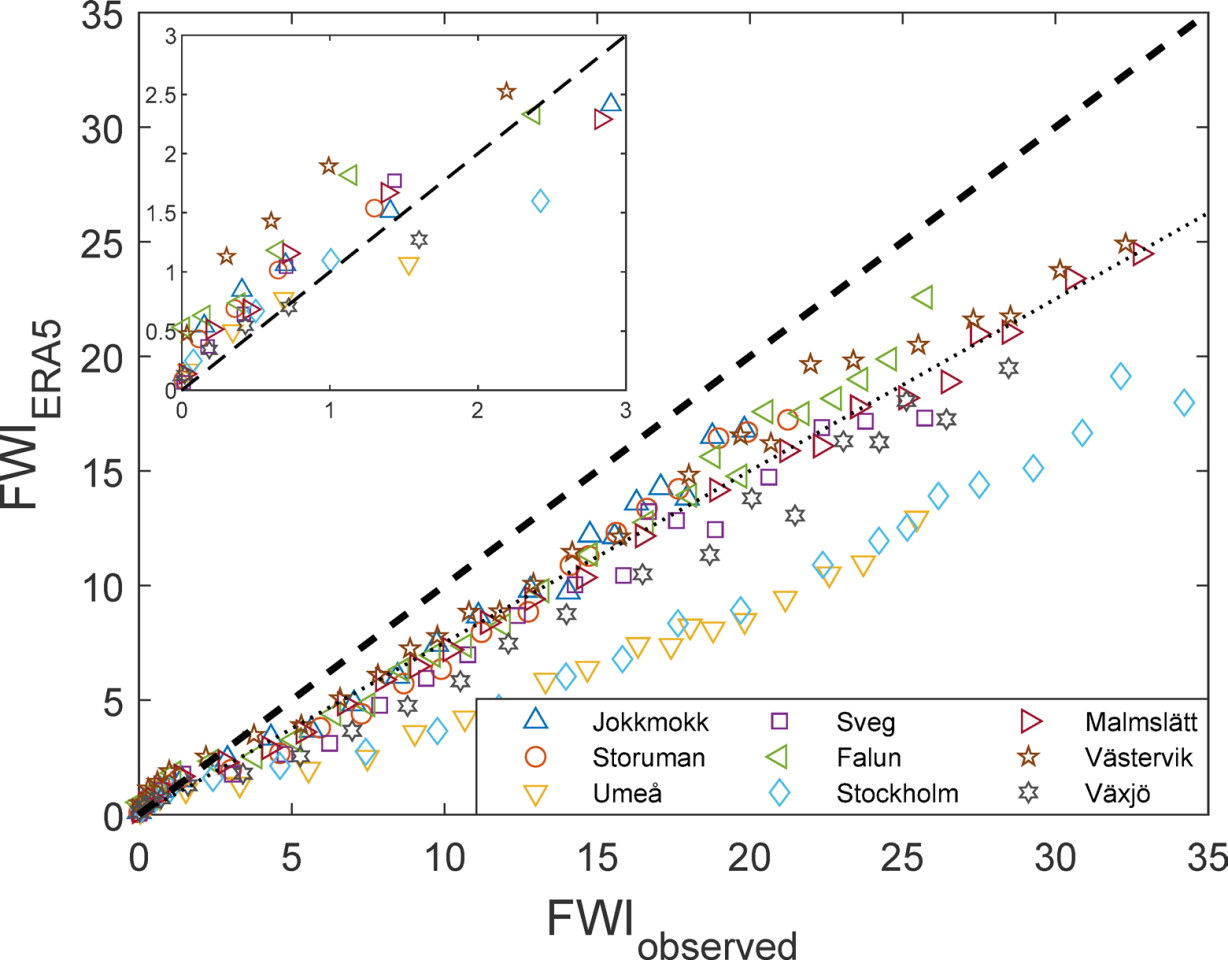
FWI-values calculated from ERA5-data (FWI_ERA5_) plotted against observation data (FWI_observed_) for all sites (1951–2020). The data is averaged over binned intervals (500 observations for FWI < 5 and 300 observations for FWI > 5) for better readability. The dashed line represents perfect correlation, and the dotted line is FWI_ERA5_ = 0.75 × FWI_observed_. The inset shows the same data for FWI < 3.

**Fig. 10:**
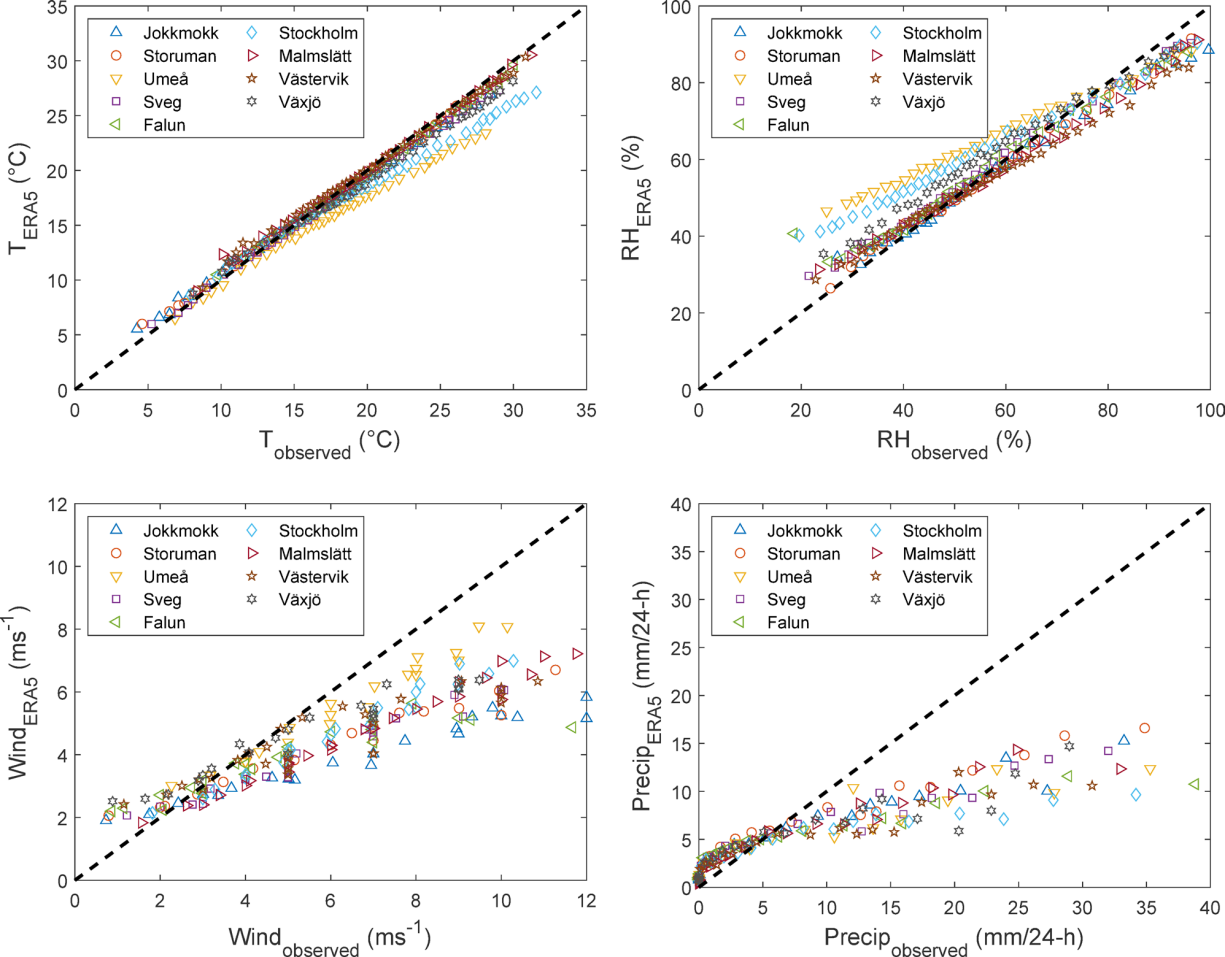
Weather input data from ERA5 plotted against observation data for all sites. The data is for each day, season and site but averaged over binned intervals for better readability. The dashed line represents perfect correlation.

The FWI-values calculated from the observation data and the ERA5 reanalysis data, respectively, showed large discrepancies for all sites. During days with FWI < 2 (representing 48% of the entire dataset), the ERA5 data generally overestimated the observational FWI-value. But for FWI > 2 the ERA5 data instead underestimated the FWI-values, and with higher fire danger the discrepancy between ERA5- and observation-based data became larger (Fig. 9). ERA5 underestimated FWI at all sites but with considerable variation; discrepancies being largest for Umeå and Stockholm. On average, ERA5-data underestimated FWI-values by 5 points for FWI = 20 and 8 points for FWI = 30, a decrease of approximately 25% (Fig. 9). This has large consequences for peak fire danger, with consistently lower FWI-7x values from ERA5-data. Additionally, the linear FWI-7x trends also differ over the 70-year period, both in absolute terms and relative to the average value of each time series such that the increasing trends from reanalysis data is between 23 and 82% lower than that from the observational data (Table 3). Similar discrepancies between observed and reanalysed data were observed for the underlying weather parameters, i.e. T, W, RH and 24-h precipitation (Fig. 10).

**Table 3:** Average values of FWI-7x and its linear trend over 70 years for each site calculated from ERA5 and from observational weather data, respectively.

Site	ERA5		Observations		Difference (ERA5-Obs.)	
	Average FWI-7x	Linear trend (per 70 years)	Average FWI-7x	Linear trend (per 70 years)	Average FWI-7x	Linear trend (per 70 years)
Jokkmokk	11.5	+ 3.89	12.5	− 1.2	− 1.0	+ 5.1*
Storuman	11.2	+ 3.26	13.5	+ 6.1	− 2.3	− 2.8
Umeå	8.6	+ 0.27	15.8	+ 0.2	− 7.2	+ 0.1
Sveg	11.1	+ 0.91	14.5	+ 2.2	− 3.4	− 1.3
Falun	13.9	+ 0.75	16.4	+ 4.0	− 2.5	− 3.3
Stockholm	11.4	+ 0.76	21.0	+ 3.8	− 9.6	− 3.0
Malmslätt	13.9	+ 1.66	17.6	+ 2.6	− 3.7	− 0.9
Västervik	15.5	+ 2.03	16.9	+ 5.7	− 1.4	− 3.7
Växjö	11.3	+ 1.68	16.2	+ 2.0	− 4.9	− 0.3

*The trend changes from positive to negative when calculating using observational rather than reanalysis data.

## Discussion

### Observational versus reanalysis data

Most long-term studies of weather or fire danger rely on atmospheric reanalysis products such as ERA5. However, our analysis shows that fire danger is systematically underestimated if based on ERA5 data compared to the direct meteorological observations and that fire danger trends of reanalysis-based time series differed substantially from those based on observational data (Table 3). For most sites, moderate to high ERA5-based FWI were approximately only 75% (± 15%) of the value based on observations (Fig. 9, dotted line), with an equivalent reduction of seasonal extremes such as FWI-7x.

Naturally, some difference between observational- and reanalysis datasets are expected. The first is a description of the weather at a single point (similar to what actually determines conditions for fire), whereas the second represents the average weather within a cell, estimated from a consistent model applied to a larger region. Thus, even if spatial resolution increases, reanalysis data will always be biased towards an underestimation of extreme values. This “oversmoothing” not only relates to precipitation, which during summer is mostly convective and inherently local, but also wind, temperature and therefore (indirectly) also relative humidity^20^ (Fig. 4). Our results show that the bias inherent in the ERA5 weather parameters translate into a substantial, almost linear, underestimation of the FWI-index in situations of moderate to high fire danger.

Even though weather station observations are standardised, using state-of-the-art instruments, long series of observation data from individual stations are also associated with idiosyncrasies. However, the overall trends from our nine stations should be sufficient to smooth out local eccentricities. Even if the variables are correctly read, all parameters have a degree of uncertainty and the largest are found for RH^42^ and wind speed^43^. Wind speed is intrinsically a robust parameter to measure but for long series, gradual changes in the surrounding can occur, such as trees outside of the open area growing taller or buildings being erected nearby. However, all data collected by the Swedish meteorological service and used in this study have been quality-reviewed and if stochastic or systematic errors were detected they were corrected before release of data to the public. Also, modern instruments have less uncertainties and all stations used here had automatic reading as from the mid-90 s.

The ERA5 model with its homogeneous distributions in time and space enables fire danger analyses of whole regions. They are therefore widely used to assess historical fire danger^12,44,45^ or as a baseline for future projections^11,14–16^. Our results suggest that such large-scale analyses should at least include an estimate of bias against a sample of observation data, particularly for periods of high fire danger. For regional studies, using datasets of finer resolution and better local bias correction would also reduce the oversmoothing^18^.

### Fire danger trends and drivers

Overall, the 70-year time series of actual observed FWI-7x constructed here point towards increasing fire danger over the 70-year period. While only three sites (Storuman, Falun and Västervik) exhibited a statistically significant (*p* < 0.05) linear trend, only one (Jokkmokk) had an opposing trend (non-significant). The average trend of ca + 3 index points over the period is difficult to compare to other parts of the circumboreal region as fire danger analyses based on observational weather data are scarce. However, using five climate models and averaging over a large part of Eastern Canada, Barnes et al^1^ produced a dataset with a linear FWI-7x trend of circa + 5 point between early 1950s and 2020. In contrast, Mäkelä et al^46^ used the Finish fire danger system^47^ and gridded monthly averages of temperature and precipitation (1908–2011) in Finland and found only non-significant trends in number of fire days, within a large inter-annual variability. Also, correlation between these monthly averages and outcome in terms of occurrence of large forest fires was poor, indicating that monthly weather averages are too coarse for assessing fire danger.

Increased FWI-7x was more influenced by changes in ISI (to 75% on average) than by BUI (25%) (Table A1). Jokkmokk was the only site with a substantial negative influence from BUI and Stockholm and Malmslätt were the only sites with a relatively larger positive contribution from BUI than from ISI. These two sites lie in the part of the country where summer drought is most common^49^, and the changes there were also driven by increased dryness of fuel components with a longer time-lag (i.e. high DMC and DC) as opposed to sites where the increasing fire danger was mostly driven by increasing FFMC. As FFMC represents moisture content in rapidly responding fine fuels^48^ it can be more easily influenced by short periods of extreme weather.

The changes in fire danger were largest in the early season of May and June. Late spring is also the time of year with the lowest noon-RH, while DMC usually do not peak until July. However, we cannot identify any clear trend towards earlier timing of peak fire danger, within the large interannual variability. The timing of peak fire danger follows very broad distributions from mid-May to mid-August (Fig. 8).

The increase in ISI was mostly due to increasing FFMC and only marginally influenced by trends in wind (Fig. 5b). The changes in FFMC were, in turn, estimated to be mostly influenced by increasing noon-temperature. All but one site had a negative contribution to fire danger from increasing water vapour pressure. Averaged over our nine sites, water vapour pressure during peak FWI exhibited an 8% increase over the 70-years study period, similar to an increase in summer water vapour pressure of 10–11% averaged for the entire country over roughly the same period (1951–2012)^42^. However, despite the increase in vapour pressure, the increasing noon temperature still results in lower relative air humidity. The lower relative humidity, in turn, leads to lower moisture content of light fuel and thus higher fire danger. This result is not unique for Sweden as decreasing relative humidity was reportedly the driver of 75% of the positive fire danger trends in a global study covering the period 1979–2020, using ERA5 reanalysis^44^.

Single meteorological parameters cannot explain trends in fire danger, which depend on the interaction of several different weather parameters and their variation over time. Malmslätt e.g., exhibited large increases in summer precipitation, and a decrease in the longest summer drought by several days over the study period (Table 1). Nevertheless, there was a substantial increase in FWI-7x for the same site, due to the counteracting effect of higher temperature, leading to increased BUI despite shorter periods without rain (Table 2).

Besides of the long-term trend of increasing fire danger, the very large inter-annual variability is also of interest. For example, 2018 has been described as a year of unprecedented fire danger throughout Sweden^50^, but seen over the full 70-year period, several years have been on par with 2018, e.g. 1955, 1969, 1983 (for southern sites) and 1994 (all sites) (Fig. 2). Our data represent a 1100 km N–S gradient throughout boreal and hemiboreal Northern Europe and as expected there was a gradually decreasing annual correspondence with increasing distance between stations (Fig. 4). However, in contrast to recent projections of future fire climate in this region^17,18^ we did not see any clear N–S difference in the observed fire danger trends, although the northernmost station (Jokkmokk) was contrasting to the rest, and the only one with decreasing fire danger. To determine if trends for northernmost Fennoscandia truly differs from areas further south, a wider network of stations will have to be analysed.

Our results should reflect the effect of ongoing human-caused climate change on fire danger. As such, the observed trends would be expected to increase with time. The most recent projection for Sweden^18^ suggest the largest increase in fire danger for the south-east of the country as well as along the Baltic sea coast, whereas our data shows more uniform changes throughout. However, Berg et al^18^ found increasing FFMC-projections, mostly driven by changes in noon-RH, to be the most significant factor for increasing fire danger in periods of high FWI-values, which corresponds to our results.

## Conclusions

We produce the first long-term dataset of fire danger in boreal Europe built on actual daily weather observations. Over the 70-year study period the annual maximum 7-day average of FWI increased by + 3 index points, seen over all nine stations. The increase was mainly driven by higher noon temperatures, which decrease relative humidity and increase drying rates. When comparing observation-based fire danger calculations with the corresponding calculations based on gridded reanalysis data (ERA5), the latter underestimated FWI-values by, on average, 25% for FWI > 5. Additionally, the observation- and reanalysis-based data show disproportional differences in fire-danger trends over the 70-year period. This highlights the need to carefully consider bias correction, and we suggest checking against observation data whenever exploring large-scale analyses of gridded reanalysis data.

## Electronic supplementary material

Below is the link to the electronic supplementary material.

Supplementary Material 1

Supplementary Material 2

## Data Availability

Data from the nine observation stations with daily weather as well as calculated moisture codes and indices are available online at 10.13140/RG.2.2.14676.31369. Annual peaks of the same variables are also available in separate files. Descriptions can be found with the supplementary information of this article.

## References

[1] Barnes, C. et al. *Climate change more than doubled the likelihood of extreme fire weather conditions in eastern Canada*. Scientific Report; 10.25561/105981 (2023).

[2] Burton, C. et al. Global burned area increasingly explained by climate change. *Nat. Clim. Change* **14**, 1186–1192. 10.1038/s41558-024-02140-w (2024).

[3] Beamen, E. Wildfires up to three times more likely due to climate change, new report claims, The independent—Wednesday 14 August; https://www.independent.co.uk/climate-change/news/wildfires-2024-athens-fires-climate-crisis-b2596068.html (2023). (Accessed on 2024-10-23).

[4] Lawson, B. D. & Armitage, O.B. *Weather Guide for the Canadian Forest Fire Danger Rating System*. (Natural Resources Canada, Canadian Forest Service, Northern Forestry Centre, 2008).

[5] Abatzoglou, J. T. & Williams, A. P. Impact of anthropogenic climate change on wildfire across western US forests. *Proc. Nat. Acad. Sci.* **113**(42), 11770–11775. 10.1073/pnas.1607171113 (2016). 27791053 10.1073/pnas.1607171113 PMC5081637

[6] Wotton, B. M., Flannigan, M. D. & Marshall, G. A. Potential climate change impacts on fire intensity and key wildfire suppression thresholds in Canada. *Environ. Res. Lett.* **12**, 095003. 10.1088/1748-9326/aa7e6e (2017).

[7] Dupuy, J. L. et al. Climate change impact on future wildfire danger and activity in southern Europe: A review. *Ann. Forest Sci.* **77**, 35. 10.1007/s13595-020-00933-5 (2020).

[8] Mansoor, S. et al. Elevation in wildfire frequencies with respect to the climate change. *J. Environ. Manag.* **301**, 113769. 10.1016/j.jenvman.2021.113769 (2022). 10.1016/j.jenvman.2021.113769 34600426

[9] Jones, M. W. et al. State of wildfires 2023–2024. *Earth Syst. Sci. Data* **16**, 3601–3685. 10.5194/essd-16-3601-2024 (2024).

[10] Jolly, W. M. et al. Climate-induced variations in global wildfire danger from 1979 to 2013. *Nat. Commun.* **6**, 7537. 10.1038/ncomms8537 (2015). 26172867 10.1038/ncomms8537 PMC4803474

[11] Abatzoglou, J. T., Williams, A. P. & Barbero, R. Global emergence of anthropogenic climate change in fire weather indices. *Geophys. Res. Lett.* **46**, 326–336. 10.1029/2018GL080959 (2019).

[12] Vitolo, C. et al. ERA5-based global meteorological wildfire danger maps. *Scient. Data* **7**, 216. 10.1038/s41597-020-0554-z (2020). 10.1038/s41597-020-0554-z PMC7341852 32636392

[13] Jones, M. W. et al. Global and regional trends and drivers of fire under climate change. *Rev. Geophys.* **60**, e2020RG000726. 10.1029/2020RG000726 (2022).

[14] Quilcaille, Y., Batibeniz, F., Ribeiro, A. F. S., Padrón, R. S. & Seneviratne, S. I. Fire weather index data under historical and shared socioeconomic pathway projections in the 6th phase of the Coupled Model Intercomparison Project from 1850 to 2010. *Earth Syst. Sci. Data* **15**, 2153–2177. 10.5194/essd-15-2153-2023 (2023).

[15] El Garroussi, S., Di Giuseppe, F. D. G., Barnard, C. & Wetterhall, F. Europe faces up to tenfold increase in extreme fires in a warming climate. *Npj Clim. Atmos. Sci.* **7**, 30. 10.1038/s41612-024-00575-8 (2024).

[16] Hetzer, J., Forrest, M., Ribalaygua, J., Prado-López, C. & Hickler, T. The fire weather in Europe: Large-scale trends towards higher danger. *Environ. Res. Let.* **19**, 084017. 10.1088/1748-9326/ad5b09 (2024).

[17] Yang, W., Gardelin, M., Olsson, J. & Bosshard, T. Multi-variable bias correction: Application of forest fire risk in present and future climate in Sweden. *Nat. Hazards Earth Syst. Sci.* **15**, 2037–2057. 10.5194/nhess-15-2037-2015 (2015).

[18] Berg, P. et al. *Framtida brandrisk—förändringar i perioder av hög brandrisk enligt FWI-modellen*. Myndigheten för samhällsskydd och beredskap, MSB2301 (2024).

[19] Hanson, C. E. et al. Modelling the impact of climate extremes: An overview of the MICE project. *Clim. Change* **81**, 163–177. 10.1007/s10584-006-9230-3 (2007).

[20] Hofstra, N., New, M. & McSweeney, C. The influence of interpolation and station network density on the distributions and trends of climate variables in gridded daily data. *Clim. Dynam.* **35**, 841–858. 10.1007/s00382-009-0698-1 (2010).

[21] Gualtieri, G. Analysing the uncertainties of reanalysis data used for wind resource assessment: A critical review. *Renew. Sust. Energ. Rev.* **167**, 112741. 10.1016/j.rser.2022.112741 (2022).

[22] Thomas, S. R., Nicolau, S., Martínez-Alvarado, O., Drew, D. J. & Bloomfield, H. C. How well do atmospheric reanalyses reproduce observed winds in coastal regions of Mexico?. *Meteorol. Appl.* **28**, e2023. 10.1002/met.2023 (2021).

[23] Singh, H. & Mohanty, M. P. Can atmospheric reanalysis datasets reproduce flood inundation at regional scales? A systematic analysis with ERA5 over Mahanadi River Basin, India. *Environ. Monit. Assess.* **195**(10), 1143. 10.1007/s10661-023-11798-2 (2023). 37667048 10.1007/s10661-023-11798-2

[24] Field, R. D. Evaluation of global fire weather database reanalysis and short-term forecast products. *Nat. Hazards Earth Syst. Sci.* **20**, 1123–1147. 10.5194/nhess-20-1123-2020 (2020).

[25] Gelaro, R. et al. The modern-era retrospective analysis for research and applications, version 2 (MERRA-2). *J. Clim.* **30**, 5419–5454. 10.1175/JCLI-D-16-0758.1 (2017). 10.1175/JCLI-D-16-0758.1 PMC6999672 32020988

[26] Hersbach, H. et al. The ERA5 global reanalysis. *Q. J. Roy. Meteor. Soc.* **146**, 1999–2049. 10.1002/qj.3803 (2020).

[27] McElhinny, M., Beckers, J. F., Hanes, C., Flannigan, M. & Jain, P. A high-resolution reanalysis of global fire weather from 1979 to 2018—Overwintering the Drought Code. *Earth Syst. Sci. Data* **12**, 1823–1833. 10.5194/essd-12-1823-2020 (2020).

[28] Phoo, W. W. et al. Fire activity and fire weather in a Lower Mekong subregion: Association, regional calibration, weather–adjusted trends, and policy implications. *Nat. Hazards* **120**, 13259–21328. 10.1007/s11069-024-06743-6 (2024).

[29] England, M. R. et al. The recent emergence of Arctic amplification. *Geophys. Res. Lett.* **48**, e2021GL094086. 10.1029/2021GL094086 (2021).

[30] Bush, E. et al. *Canada’s Changing Climate Report-Executive Summary*. (Government of Canada, 2019). https://changingclimate.ca/site/assets/uploads/sites/2/2019/03/CCCR_ExecSummary.pdf (Accessed October 16th, 2024).

[31] Luomaranta, A. M., Aalto, J. & Jylhä, K. Snow cover trends in Finland over 1961–2014 based on gridded snow depth observations. *Int. J. Climatol.* **39**, 3147–3159. 10.1002/joc.6007 (2019).

[32] Schimanke. S. et al. *Observerad klimatförändring i Sverige 1860–2021*. *Klimatologi* vol. 69. (Norrköping, 2022). https://www.smhi.se/polopoly_fs/1.189743!/Klimatologi_69%20Observerad%20klimatf%C3%B6r%C3%A4ndring%20i%20Sverige%2018602021.pdf (Accesses 2024-11-12).

[33] Wern, L. *Snödjup i Sverige 1904/05–2013/14*. SMHI: Norrköping (2015).

[34] Andersson, S., Bärring, L., Landelius, T., Samuelsson, P. & Schimanke, S. *SMHI Gridded Climatology, Technical Report 118*. (SMHI, 2021). https://orcid.org/0000-0003-4387-6232

[35] Magnus, G. Versuche über die Spannkräfte des Wasserdampfs. *Ann. Phys. Berlin* **137**, 225–247. 10.1002/andp.18441370202 (1844).

[36] Swedish Meteorological and Hydrological Institute (SMHI). *Ladda ner meteorologiska observationer* https://www.smhi.se/data/meteorologi/ladda-ner-meteorologiska-observationer (2023). (visited 24 November 24th, 2023).

[37] Turner, J. A. & Lawson, B. D. *Weather in the Canadian Forest Fire Danger Rating System: A User Guide to National Standards and Practices*. Information Report BC-X-177. 40. (Environment Canada, Canadian Forestry Service, Pacific Forest Research Centre, 1978).

[38] Sjöström, J. & Granström, A. A phenology-driven fire danger index for northern grasslands. *Int. J. Wildland Fire* **32**, 1332–1346. 10.1071/WF23013 (2023).

[39] Sjöström, J. & Granström, A. Human activity and demographics drive the fire regime in a highly developed European boreal region. *Fire Saf. J.* **136**, 103743. 10.1016/j.firesaf.2023.103743 (2023).

[40] Van Wagner, C. E. & Pickett, T. L*. Equations and FORTRAN Program for the Canadian Forest Fire Weather Index System*. Forestry Technical Report 33. (Canadian Forest Service, 1985).

[41] van Oldenborgh, G. J. et al. Attribution of the Australian bushfire risk to anthropogenic climate change. *Nat. Hazards Earth Syst. Sci.* **21**, 941–960. 10.5194/nhess-21-941-2021 (2021).

[42] Wern, L. Luftfuktighet-Variationer i Sverige. In *Meteorologi* vol. 154. (SMHI, 2013).

[43] Wern, L. & Bärring, L. Sveriges vindklimat 1901–2008: Analys av förändring i geostrofisk vind. In *Meteorologi* vol. 138. (SMHI, 2009).

[44] Jain, P., Castellanos-Acuna, D., Coogan, S. C., Abatzoglou, J. T. & Flannigan, M. D. Observed increases in extreme fire weather driven by atmospheric humidity and temperature. *Nat. Clim. Change* **12**, 63–70. 10.1038/s41558-021-01224-1 (2022).

[45] Baijnath-Rodino, J. A., Le, P. V. V., Foufoula-Georgiou, E. & Banerjee, T. Historical spatiotemporal changes in fire danger potential across biomes. *Sci. Total Environ.* **870**, 161954. 10.1016/j.scitotenv.2023.161954 (2023). 36736401 10.1016/j.scitotenv.2023.161954

[46] Mäkelä, H. M., Laapas, M. & Venäläinen, A. Long-term temporal changes in the occurrence of a high forest fire danger in Finland. *Nat. Hazard. Earth Syst. Sci.* **12**, 2591–2601. 10.5194/nhess-12-2591-2012 (2012).

[47] Vajda, A., Venäläinen, A., Suomi, I., Junila, P. & Mäkelä, H. M. Assessment of forest fire danger in a boreal forest environment: Description and evaluation of the operational system applied in Finland. *Meteorol. Appl.* **21**, 879–887. 10.1002/met.1425 (2014).

[48] Van Wagner, C. E. *A Method of Computing Finefuel Moisture content Throughout the Diurnal Cycle*. Fish. and Environ. Can., Can. For. Serv., Petawawa For. Exp. Stn., Chalk River, Ont., Inf. Rep. PS-X-69 (1977).

[49] Ångström, A. Some characteristics of the climate of Stockholm. *Geogr. Ann. A* **14**, 165–196 (1932).

[50] Björheden, R. & Johannesson, T. *Effekter på svenskt skogsbruk av sommaren 2018*. Skogforsk Arbetsrapport 1012 https://www.skogforsk.se/cd_20190502151614/contentassets/8020a8a5edd645c5a7352e5280853eef/arbetsrapport-1012-2019.pdf (2018). (Accessed on 2024-10-31).

