# Supplementary material for: 70 Years of observational weather data show increasing fire danger for boreal Europe and reveal bias of ERA5 reanalysed data
Source: Sci Rep. 2025 Jun 20;15:20111. doi: 10.1038/s41598-025-04200-3 (PMC12181286; doi:10.1038/s41598-025-04200-3)
Supplement: Supplementary file 1 — Supplementary Material 1 [file 41598_2025_4200_MOESM1_ESM.pdf]

Supplementary information to

70 years of observational weather data show increasing fire danger for boreal Europe and reveal bias of ERA5 reanalysed data

Johan Sjöström, Frida Vermina Plathner, Anders Granström

Scientific Reports

Content

1. Description of the associated datasets 2
2. End of snow period and timing of FWI-7x 4
3. Correlation between the FWI-7x for different sites 6
4. Model for attributing changes in FWI-7x to changes in weather parameters 7

1. Description of the associated datasets

Supplementary to the article these is a zip-file with the data produced in the study. For each site, there is one file with daily values from snow melt to October 31st and one file with seasonal characteristics (such as FWI-7x). We exemplify the structure of the data (xlsx-files) below.

Download the data from https://doi.org/10.13140/RG.2.2.14676.31369

Supplementary Table S1. Weather and calculated fire danger for each day during the season (1951-2020).

Name	'FWI Vastervik 1951-2020.xlsx'
Description	Weather and fire danger every day (1951 - 2020) from snow melt to Oktober 31st for Västervik
Column 1(A)	Date (YYYYMMDD)
Column 2(B)	Year
Column 3(C)	Month
Column 4(D)	Day
Column 5(E)	Ordinal day
Column 6(F)	Temperature 12:00 UTC
Column 7(G)	RH (%)
Column 8(H)	10-m open wind speed 12.00 UTC (ms-1)
Column 9(I)	Rain (mm/24h)
Column 10(J)	FFMC
Column 11(K)	m (%) – The moisture level of the fine fuel
Column 12(L)	DMC
Column 13(M)	M (%) – The moisture level of the duff layer
Column 14(N)	DC
Column 15(O)	Q – The equivalent moisture level of deep layers
Column 16(P)	ISI
Column 17(Q)	BUI
Column 18(R)	FWI
Column 19(S)	Ordinal day/365
Column 20(T)	DRS
Column 21(U)	DMC7 (7-days DMC average)
Column 22(V)	FWI7 (7-days FWI average)
Column 23(W)	DSR7 (7-days FWI average)

Supplementary Table S2. Characteristic values for each season 1951-2020

Name	'Annuals full Vastervik 1951-2020.xlsx'
Description	Characteristic values for weather and fire danger indices (1951 - 2020) for Västervik
Column 1(A)	Year
Column 2(B)	Average Temp at 12.00. For June 1st – August 31st (JJA) (°C)
Column 3(C)	T7x (°C)
Column 4(D)	RH at 12.00 UTC (Average JJA) (%)
Column 5(E)	RH7x
Column 6(F)	10-m open wind speed 12.00 UTC (Average JJA) (ms^{-1})
Column 7(G)	Max 7-day aver. wind speed when also FFMC>85 & DMC>20 (ms^{-1})
Column 8(H)	Total rain (JJA) (mm)
Column 9(I)	Number of days with rain (JJA)
Column 10(J)	Number of days with >0.5 mm/24h (JJA)
Column 11(K)	Longest dryspell (days)
Column 12(L)	Longest dryspell with <0.5 mm/24h (days)
Column 13(M)	DRSx
Column 14(N)	DRS7x
Column 15(O)	SSR (JJA)
Column 16(P)	SSR (snow melt to October 31st)
Column 17(Q)	FWIx
Column 18(R)	FWI7x
Column 19(S)	Average FWI (JJA)
Column 20(T)	DMCx
Column 21(U)	DMC7x
Column 22(V)	Average DMC (JJA)
Column 23(W)	FFMCx
Column 24(X)	FFMC7x
Column 25(Y)	Average FFMC (JJA)
Column 26(Z)	ISIx
Column 27(AA)	ISI7x
Column 28(AB)	Average ISI (JJA)
Column 29(AC)	DCx
Column 30(AD)	DC7x
Column 31(AE)	Average DC (JJA)
Column 32(AF)	BUIx
Column 33(AG)	BUI7x
Column 34(AH)	Average BUI (JJA)
Column 35(AI)	Number of days with (FWI<11) (JJA)
Column 36(AJ)	Number of days with (FWI>20) (JJA)
Column 37(AK)	Number of days with DMC<10 (JJA)
Column 38(AL)	Number of days with DMC>40 (JJA)

The equivalent files are available for all sites.

Data is free to use if cited accordingly and can be downloaded from https://doi.org/10.13140/RG.2.2.14676.31369

2. End of snow period and timing of FWI-7x

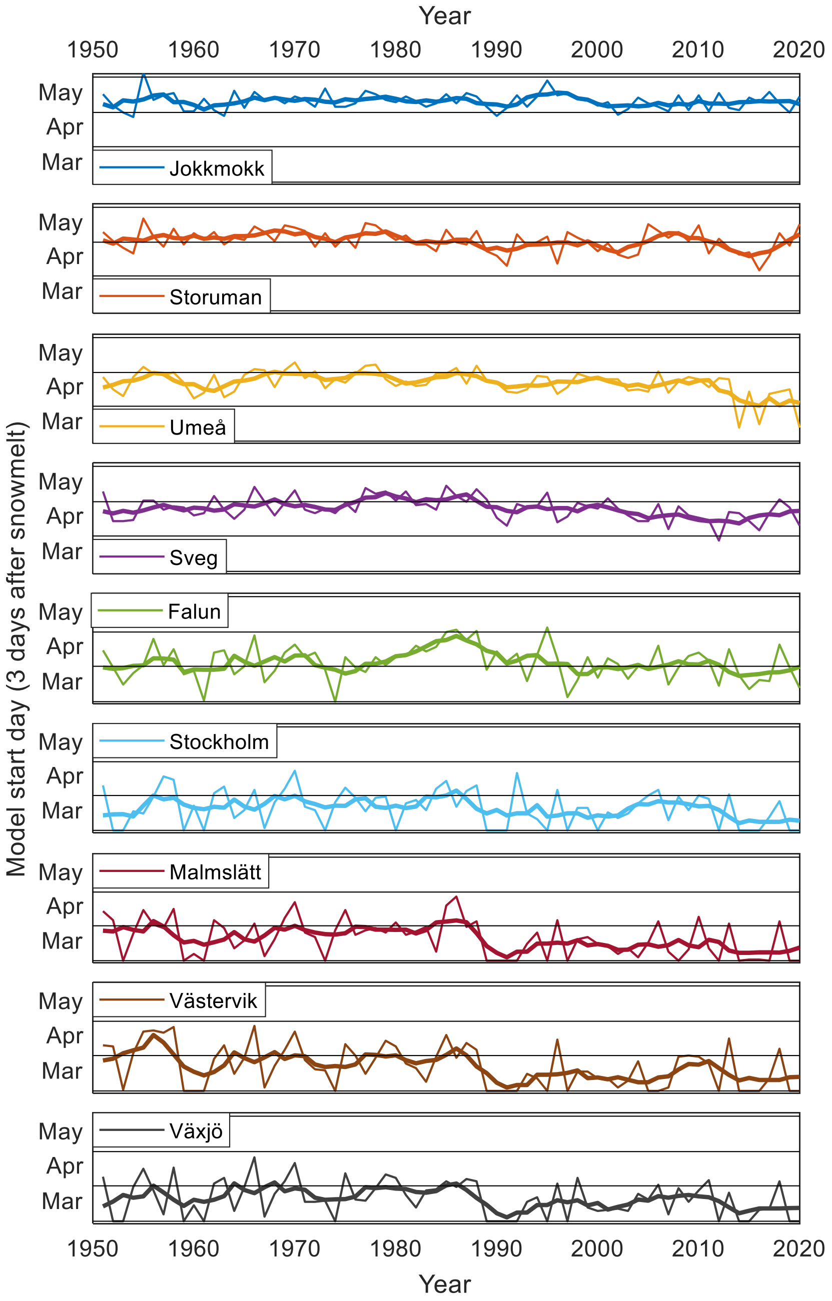

Supplementary Figure S1. The start day of the model run for each site and year. Thick lines are 5-years moving average of the annual data.

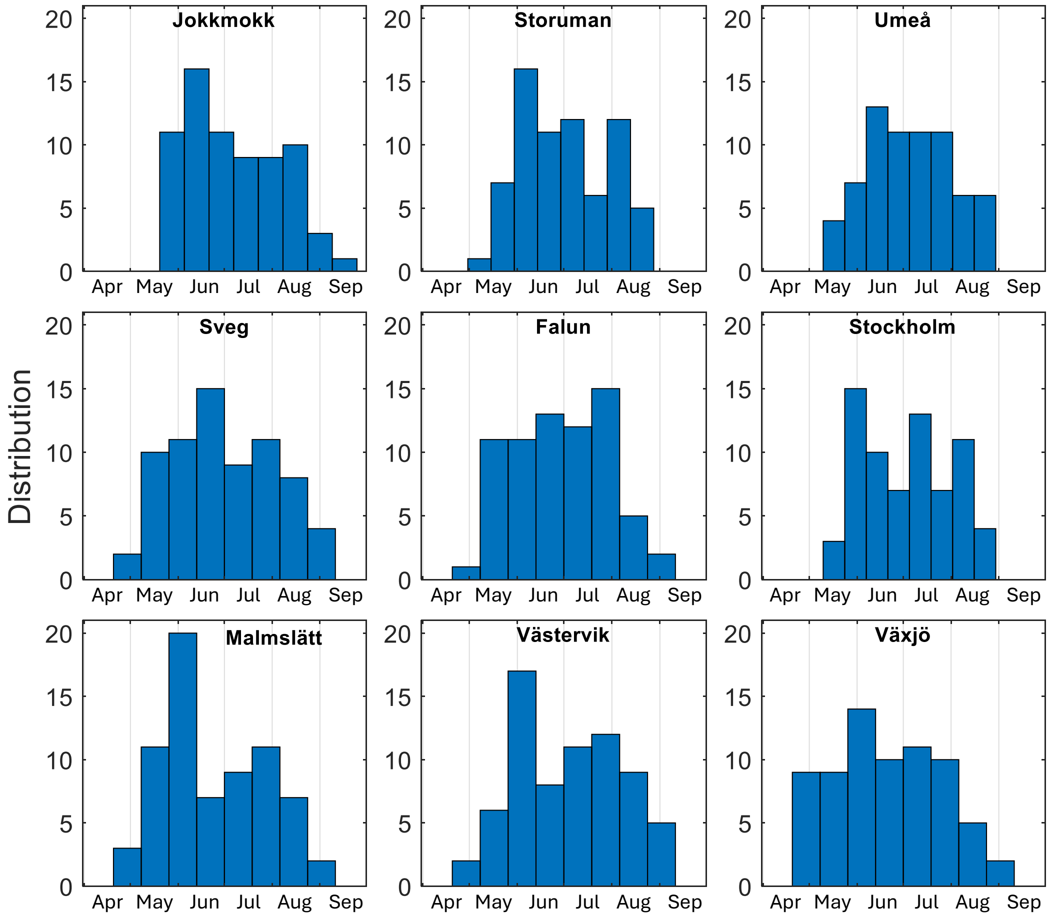

Supplementary Figure S2. Histograms of the seasonal timing of maximum fire danger, FWI-7x, at the different sites over the period 1951-2020. All distributions are plotted with eight bins.

3. Correlation between the FWI-7x for different sites

Supplementary Table S3. Pearson cross correlation matrix $\left(\rho_{i,j}\right)$ for FWI-7x between the sites, arranged from North to South.

	Jokkmokk	Storuman	Umeå	Sveg	Falun	Stockholm	Malmslätt	Västervik	Växjö
Jokkmokk	1.00	0.48	0.50	0.40	0.37	0.44	0.26	0.26	0.32
Storuman	0.48	1.00	0.53	0.61	0.48	0.36	0.28	0.44	0.40
Umeå	0.50	0.53	1.00	0.45	0.57	0.45	0.38	0.36	0.47
Sveg	0.40	0.61	0.45	1.00	0.67	0.50	0.45	0.49	0.47
Falun	0.37	0.48	0.57	0.67	1.00	0.68	0.59	0.60	0.57
Stockholm	0.44	0.36	0.45	0.50	0.68	1.00	0.71	0.70	0.55
Malmslätt	0.26	0.28	0.38	0.45	0.59	0.71	1.00	0.76	0.66
Västervik	0.26	0.44	0.36	0.49	0.60	0.70	0.76	1.00	0.76
Växjö	0.32	0.40	0.47	0.47	0.57	0.55	0.66	0.76	1.00

	$\rho_{i,j} < 0.4$
	$0.4 < \rho_{i,j} < 0.5$
	$0.5 < \rho_{i,j} < 0.6$
	$0.6 < \rho_{i,j} < 0.7$
	$\rho_{i,j} > 0.7$

4. Model for attributing changes in FWI-7x to changes in weather parameters

Supplementary Table S4. Nomenclature

**Variable**	**Label**	**Description**
BUI-7_m	Concurrent BUI	7-days average of the BUI index during the same period of FWI-7x, the maximum 7-days average of FWI
bui_y	Linear bui trend	Linear fit over the years (y) of each season's BUI-7_m
DC-7_m	Concurrent DC	7-days average of the DC code value during the same period of FWI-7x, the maximum 7-days average of FWI
dc_y	Linear dc trend	Linear fit over the years (y) of each season's DC-7_m
DMC-7_m	Concurrent DMC	7-days average of the DMC code value during the same period of FWI-7x, the maximum 7-days average of FWI
dmc_y	Linear dmc trend	Linear fit over the years (y) of each season's DMC-7_m
FFMC-7_m	Concurrent FFMC	7-days average of the FFMC code value during the same period of FWI-7x, the maximum 7-days average of FWI
$ffmc_y$	Linear ffmc trend	Linear fit over the years (y) of each season's FFMC-7_m
FWI-7X	Seasonal characteristic	Maximum 7-days average of the FWI index value during one season
fwi_y	Linear fwi trend	Linear fit over the years (y) of annual FWI-7X values
ISI-7_m	Concurrent ISI	7-days average of the ISI index during the same period of FWI-7x, the maximum 7-days average of FWI
isi_y	Linear bui trend	Linear fit over the years (y) of each season's ISI-7_m
LM_{BUI}	Linear regression of BUI	Linear regression fit of annual BUI-7_m to concurrent moisture codes (DMC-7_m and DC-7_m)
LM_{DC}	Linear regression of DC	Linear regression fit of annual DC-7_m to DC-rain and Tav (P_{DC} and T_{av})
LM_{DMC}	Linear regression of DMC	Linear regression fit of annual DMC-7_m to DMC-rain and concurrent temperature and water vapour pressure (T-7_m / vp-7_m)
LM_{FFMC}	Linear regression of FFMC	Linear regression fit of annual FFMC-7_m to concurrent temperature, water vapour pressure and wind (T-7_m / vp-7_m / W-7_m)
LM_{FWI}	Linear regression of FWI	Linear regression fit of annual FWI-7x to concurrent subindices (ISI-7_m and BUI-7_m)
LM_{ISI}	Linear regression of ISI	Linear regression fit of annual ISI-7_m to concurrent FFMC and wind (FFMC-7_m and W-7_m)
$\mathrm{P}_{DC}(y)$	DC-rain	Cumulative rain of year *y* from start of the season to maximum of FWI-7 subtracted by 2.8 mm/24-h and a weight of53 days characteristic exponential decline
p_{dcy}	Linear regression of $\mathrm{P}_{DC}(y)$	Linear fit over the years (y) of each season's $\mathrm{P}_{DC}(y)$
$\mathrm{P}_{DMC}(y)$	DMC-rain	Cumulative rain of year *y* from start of the season to maximum of FWI-7 subtracted by 1.5 mm/24-h and a weight of 15 days characteristic exponential decline
p_{dmcy}	Linear regression of $\mathrm{P}_{DMC}(y)$	Linear fit over the years (y) of each season's $\mathrm{P}_{DMC}(y)$
T-7_m	Concurrent temp	7-days average of noon temperature during the same period of FWI-7x, the maximum 7-days average of FWI
t_y	Linear temp trend	Linear fit over the years (y) of each season's T-7_m
Tav	Average temp	Average temperature from start of season to day of maximum FWI-7
t_{avy}	Linear Tav trend	Linear fit over the years (y) of each season's Tav
vp-7_m	Concurrent water vapour pressure	7-days average of the water vapour pressure during the same period of FWI-7x, the maximum 7-days average of FWI
vp_{wy}	Linear vp trend	Linear fit over the years (y) of each season's vp-7_m

W-7_m	Concurrent wind	7-days average of the wind speed during the same period of FWI-7x, the maximum 7-days average of FWI
w_y	Linear wind trend	Linear fit over the years (y) of each season's W-7_m
Δ^{bui}_{dmc}, Δ^{bui}_{dc}		The absolute contribution from dmc_y and dc_y, respectively, to the linear change over 70 years in bui_y
Δ^{bui}_{res}		Residual change in bui_y over 70 years not captured by Δ^{bui}_{dmc} and Δ^{bui}_{dc}
Δ^{dc}_{Pdc}, Δ^{dc}_{Tav}		The absolute contribution from p_{dcy} and t_{avy}, respectively, to the linear change over 70 years in dc_y
Δ^{dc}_{res}		Residual change in dc_y over 70 years not captured by Δ^{dc}_{Pdc} and Δ^{dc}_{Tav}
Δ^{dmc}_{vp}, Δ^{dmc}_{t}, Δ^{dmc}_{Pdmc}		The absolute contribution from vp_{wy}, t_y and p_{dmcy}, respectively, to the linear change over 70 years in dmc_y
Δ^{dmc}_{res}		Residual change in dmc_y over 70 years not captured by Δ^{dmc}_{vp}, Δ^{dmc}_{t} and Δ^{dmc}_{Pdmc}
Δ^{ffmc}_{vp}, Δ^{ffmc}_{t}, Δ^{ffmc}_{w}		The absolute contribution from vp_{wy}, t_y and w_y, respectively, to the linear change over 70 years in $ffmc_y$
Δ^{ffmc}_{res}		Residual change in $ffmc_y$ over 70 years not captured by Δ^{ffmc}_{vp}, Δ^{ffmc}_{t} and Δ^{ffmc}_{w}
Δ^{fwi}_{isi}, Δ^{fwi}_{bui}		The absolute contribution from isi_y and bui_y, respectively, to the linear change over 70 years in FWI-7x (change in fwi_y)
Δ^{fwi}_{res}		Residual change in FWI-7x over 70 years not captured by Δ^{fwi}_{isi} and Δ^{fwi}_{bui}
Δ^{isi}_{ffmc}, Δ^{isi}_{w}		The absolute contribution from $ffmc_y$ and w_y, respectively, to the linear change over 70 years in isi_y
Δ^{isi}_{res}		Residual change in isi_y over 70 years not captured by Δ^{isi}_{ffmc}, and Δ^{isi}_{w}

Model description

FWI-7x (Trends distributed between Δ^{fwi}_{isi}, Δ^{fwi}_{isi} and Δ^{fwi}_{res})

- Extract annual 7-days average of ISI and BUI during the 7 days of maximum FWI (ISI-7_m and BUI-7_m)
- Fit a linear regression of FWI-7x to the predictor variables ISI-7_m and BUI-7_m
  - $\text{LM}_{FWI} = \beta_0 + \beta_1 \text{ISI-}7_m + \beta_2 \text{BUI-}7_m$
- Attribute the changes in FWI-7x from ISI and BUI using the linear trends in subindices
  - $\Delta^{fwi}_{isi} = \beta_1(isi_{2020} - isi_{1951})$
  - $\Delta^{fwi}_{bui} = \beta_2(bui_{2020} - bui_{1951})$
  - $\Delta^{fwi}_{res} = fwi_{2020} - fwi_{1951} - \Delta^{fwi}_{isi} - \Delta^{fwi}_{bui}$

ISI (Trends distributed between Δ^{isi}_{ffmc}, Δ^{isi}_{w} and Δ^{isi}_{res})

- Extract annual 7-days average of FFMC and wind during the 7 days of maximum FWI (FFMC-7_m and W-7_m)
- Fit a linear regression of ISI-7_m to the predictor variables FFMC-7_m and W-7_m
  - $\text{LM}_{ISI} = \beta_0 + \beta_1 \text{FFMC-}7_m + \beta_2 \text{W-}7_m$
- Attribute the changes in ISI-7_m from FFMC and wind using their linear trends
  - $\Delta^{isi}_{ffmc} = \beta_1(ffmc_{2020} - ffmc_{1951})$
  - $\Delta^{isi}_{w} = \beta_2(w_{2020} - w_{1951})$
  - $\Delta^{isi}_{res} = isi_{2020} - isi_{1951} - \Delta^{isi}_{ffmc} - \Delta^{isi}_{w}$

BUI (Trends distributed between Δ_{dmc}^{bui}, Δ_{dc}^{bui} and Δ_{res}^{bui})

- Extract annual 7-days average of DMC and DC during the 7 days of maximum FWI (DMC-7_m and DC-7_m)
- Fit a linear regression of BUI-7_m to the predictor variables DMC-7_m and DC-7_m
  - $\mathrm{LM}_{BUI} = \beta_0 + \beta_1 \mathrm{DMC\text{-}7}_m + \beta_2 \mathrm{DC\text{-}7}_m$
- Attribute the changes in BUI-7_m from DMC and DC using their linear trends
  - $\Delta_{dmc}^{bui} = \beta_1(dmc_{2020} - dmc_{1951})$
  - $\Delta_{dc}^{bui} = \beta_2(dc_{2020} - dc_{1951})$
  - $\Delta_{res}^{bui} = bui_{2020} - bui_{1951} - \Delta_{dmc}^{bui} - \Delta_{dc}^{bui}$

FFMC (Trends distributed between Δ_{t}^{ffmc}, Δ_{vp}^{ffmc}, Δ_{w}^{ffmc} and Δ_{res}^{ffmc})

- Extract annual 7-days average of temperature, water vapour pressure and wind during the 7 days of maximum FWI (T-7_m, vp-7_m and W-7_m)
- Fit a linear regression of FFMC-7_m to the predictor variables T-7_m, vp-7_m and W-7_m
  - $\mathrm{LM}_{FFMC} = \beta_0 + \beta_1 \mathrm{vp\text{-}7}_m + \beta_2 \mathrm{T\text{-}7}_m + \beta_3 \mathrm{W\text{-}7}_m$
- Attribute the changes in FFMC-7_m from temperature, water vapour pressure and wind using their linear trends
  - $\Delta_{vp}^{ffmc} = \beta_1(vp_{w,2020} - vp_{w,1951})$
  - $\Delta_{t}^{ffmc} = \beta_2(t_{2020} - t_{1951})$
  - $\Delta_{res}^{ffmc} = ffmc_{2020} - ffmc_{1951} - \Delta_{vp}^{ffmc} - \Delta_{t}^{ffmc}$

DMC (Trends distributed between Δ_{t}^{dmc}, Δ_{vp}^{dmc}, Δ_{Pdmc}^{dmc} and Δ_{res}^{dmc})

- Extract annual 7-days average of temperature, water vapour pressure and DMC-rain (P_{DMC}) during the 7 days of maximum FWI (T-7_m, vp-7_m and P_{DMC})
- Fit a linear regression of DMC-7_m to the predictor variables T-7_m, vp-7_m and W-7_m
  - $\mathrm{LM}_{DMC} = \beta_0 + \beta_1 \mathrm{vp\text{-}7}_m + \beta_2 \mathrm{T\text{-}7}_m + \beta_3 \mathrm{P}_{DMC}$
- Attribute the changes in DMC-7_m from temperature, water vapour pressure and DMC-rain using their linear trends
  - $\Delta_{vp}^{dmc} = \beta_1(vp_{w,2020} - vp_{w,1951})$
  - $\Delta_{t}^{dmc} = \beta_2(t_{2020} - t_{1951})$
  - $\Delta_{res}^{dmc} = dmc_{2020} - dmc_{1951} - \Delta_{vp}^{dmc} - \Delta_{t}^{dmc}$

DC (Trends distributed between Δ_{t}^{dc} and Δ_{prec}^{dc})

- Extract the average temperature of the season from start to the day of maximum 7-days average FWI and the characteristic DC-absorbed precipitation, DC-rain (Tav and P_{DC})
- Fit a linear regression of DC-7_m to the predictor variables Tav and P_{DC})
  - $\mathrm{LM}_{DC} = \beta_0 + \beta_1\, Tav + \beta_2 \mathrm{P}_{DC}$
- Attribute the changes in DC-7_m from temperature and precipitation using their linear trends
  - $\Delta_{t}^{dc} = \beta_1(t_{av,2020} - t_{av,1951})$

- $\Delta_{prec}^{dc} = \beta_2(p_{dc,2020} - p_{dc,1951})$
- $\Delta_{res}^{dc} = dc_{2020} - dc_{1951} - \Delta_t^{dc} - \Delta_{prec}^{dc}$

Multivariate linear regression models of indices and moisture codes

$\mathrm{LM}_{FWI} = \beta_0 + \beta_1 \mathrm{ISI\text{-}7}_m + \beta_2 \mathrm{BUI\text{-}7}_m$

	Intercept		ISI-7$_m$		BUI-7$_m$	
	β_0	*p*-value	β_1	*p*-value	β_2	*p*-value
Jokkmokk	-4.03	<0.01	2.20	<0.01	0.12	<0.01
Storuman	-4.61	<0.01	2.23	<0.01	0.13	<0.01
Umeå	-4.75	<0.01	2.40	<0.01	0.12	<0.01
Sveg	-4.31	<0.01	2.11	<0.01	0.13	<0.01
Falun	-4.74	<0.01	2.34	<0.01	0.12	<0.01
Stockholm	-4.46	<0.01	2.50	<0.01	0.11	<0.01
Malmslätt	-4-85	<0.01	2.47	<0.01	0.12	<0.01
Västervik	-3.81	<0.01	2.58	<0.01	0.10	<0.01
Växjö	-3.57	<0.01	2.26	<0.01	0.11	<0.01

$\mathrm{LM}_{ISI} = \beta_0 + \beta_1 \mathrm{FFMC\text{-}7}_m + \beta_2 \mathrm{W\text{-}7}_m$

	Intercept		FFMC-7$_m$		W-7$_m$	
	β_0	*p*-value	β_1	*p*-value	β_2	*p*-value
Jokkmokk	-36.8	<0.01	0.46	<0.01	0.18	<0.01
Storuman	-46.1	<0.01	0.56	<0.01	0.16	<0.01
Umeå	-47.0	<0.01	0.57	<0.01	0.22	<0.01
Sveg	-43.4	<0.01	0.54	<0.01	0.16	<0.01
Falun	-59.6	<0.01	0.71	<0.01	0.29	<0.01
Stockholm	-65.9	<0.01	0.78	<0.01	0.27	<0.01
Malmslätt	-60.0	<0.01	0.71	<0.01	0.22	<0.01
Västervik	-59.9	<0.01	0.71	<0.01	0.29	<0.01
Växjö	-49.2	<0.01	0.60	<0.01	0.22	<0.01

$\mathrm{LM}_{BUI} = \beta_0 + \beta_1 \mathrm{DMC\text{-}7}_m + \beta_2 \mathrm{DC\text{-}7}_m$

	Intercept		DMC-7$_m$		DC-7$_m$	
	β_0	*p*-value	β_1	*p*-value	β_2	*p*-value
Jokkmokk	-2.48	<0.01	0.90	<0.01	0.090	<0.01
Storuman	0.14	0.87	0.83	<0.01	0.092	<0.01
Umeå	-0.98	0.33	0.87	<0.01	0.088	<0.01
Sveg	0.01	0.99	0.86	<0.01	0.084	<0.01
Falun	-1.60	0.09	0.88	<0.01	0.087	<0.01
Stockholm	-2.47	<0.01	0.84	<0.01	0.098	<0.01
Malmslätt	-1.57	0.05	0.83	<0.01	0.099	<0.01
Västervik	-2.16	0.06	0.89	<0.01	0.087	<0.01
Växjö	-0.62	0.32	0.83	<0.01	0.095	<0.01

$\mathrm{LM}_{FFMC} = \beta_0 + \beta_1 \mathrm{vp\text{-}7}_m + \beta_2 \mathrm{T\text{-}7}_m + \beta_3 \mathrm{W\text{-}7}_m$

	Intercept		vp-7_m		T-7_m		W-7_m	
	β_0	*p*-value	β_1	*p*-value	β_2	*p*-value	β_3	*p*-value
Jokkmokk	84.8	<0.01	-58.6	<0.01	0.62	<0.01	0.056	0.55
Storuman	83.9	<0.01	-54.6	<0.01	0.65	<0.01	0.017	0.86
Umeå	84.1	<0.01	-46.1	<0.01	0.58	<0.01	0.041	0.74
Sveg	85.2	<0.01	-50.6	<0.01	0.54	<0.01	0.080	0.62
Falun	83.8	<0.01	-46.4	<0.01	0.60	<0.01	0.021	0.84
Stockholm	82.5	<0.01	-44.9	<0.01	0.61	<0.01	0.023	0.04
Malmslätt	82.7	<0.01	-44.8	<0.01	0.61	<0.01	0.021	0.01
Västervik	83.5	<0.01	-48.7	<0.01	0.63	<0.01	0.034	0.62
Växjö	83.5	<0.01	-56.4	<0.01	0.67	<0.01	0.10	0.40

$\mathrm{LM}_{DMC} = \beta_0 + \beta_1 \mathrm{vp\text{-}7}_m + \beta_2 \mathrm{T\text{-}7}_m + \beta_3 \mathrm{P}_{DMC}$

	Intercept		vp-7_m		T-7_m		P_{DMC}	
	β_0	*p*-value	β_1	*p*-value	β_2	*p*-value	β_3	*p*-value
Jokkmokk	27.3	<0.01	-44.4	0.67	1.22	0.14	-1.46	<0.01
Storuman	17.1	0.03	-118	0.15	3.02	<0.01	-1.37	<0.01
Umeå	36.7	<0.01	-152	0.06	3.26	<0.01	-3.47	<0.01
Sveg	13.6	0.14	-143	0.09	3.28	<0.01	-1.41	<0.01
Falun	17.2	0.09	-58.6	0.31	3.02	<0.01	-2.29	<0.01
Stockholm	15.9	0.13	-149	0.02	4.24	<0.01	-3.59	<0.01
Malmslätt	-12.1	0.31	-160	0.04	5.28	<0.01	-2.72	<0.01
Västervik	-0.92	0.95	-254	0.01	6.11	<0.01	-4.37	<0.01
Växjö	-36.3	0.02	-225	0.07	6.87	<0.01	-2.81	<0.01

$\mathrm{LM}_{DC} = \beta_0 + \beta_1 \mathit{Tav} + \beta_2 \mathrm{P}_{DC}$

	Intercept		Tav		P_{DC}	
	β_0	*p*-value	β_1	*p*-value	β_2	*p*-value
Jokkmokk	-164	<0.01	17.4	<0.01	-2.71	<0.01
Storuman	-205	<0.01	20.1	<0.01	-2.66	<0.01
Umeå	-254	<0.01	23.2	<0.01	-2.77	<0.01
Sveg	-194	<0.01	19.6	<0.01	-2.48	<0.01
Falun	-278	<0.01	21.7	<0.01	-3.14	<0.01
Stockholm	-339	<0.01	23.4	<0.01	-3.43	<0.01
Malmslätt	-308	<0.01	24.5	<0.01	-2.98	<0.01
Västervik	-316	<0.01	24.5	<0.01	-3.01	<0.01
Växjö	-287	<0.01	26.0	<0.01	-2.56	<0.01

Linear trends of annual values of indices and codes

$fwi_y = \beta_0 + \beta_1 y$

	Intercept		year (y)	
	β_0	*p*-value	β_1	*p*-value
Jokkmokk	46.6	<0.01	-0.017	0.73
Storuman	-163	0.80	0.089	<0.01
Umeå	8.84	<0.01	0.0035	0.69
Sveg	-48.5	0.02	0.032	0.11
Falun	-98.9	0.16	0.058	<0.01
Stockholm	-87.1	0.01	0.055	0.06
Malmslätt	-57.7	<0.01	0.038	0.26
Västervik	-148	0.58	0.083	<0.01
Växjö	-41.9	<0.01	0.029	0.63

$isi_y = \beta_0 + \beta_1 y$

	Intercept		year (y)	
	β_0	*p*-value	β_1	*p*-value
Jokkmokk	80.7	<0.01	0.0038	0.73
Storuman	-5.11	0.80	0.047	<0.01
Umeå	96.7	<0.01	-0.0038	0.69
Sveg	53.6	0.02	0.018	0.11
Falun	24.7	0.16	0.033	<0.01
Stockholm	51.3	0.01	0.020	0.06
Malmslätt	67.2	<0.01	0.011	0.26
Västervik	10.1	0.58	0.040	<0.01
Växjö	80.4	<0.01	0.0047	0.63

$bui_y = \beta_0 + \beta_1 y$

	Intercept		year (y)	
	β_0	*p*-value	β_1	*p*-value
Jokkmokk	427	0.12	-0.19	0.17
Storuman	-364	0.11	0.21	0.07
Umeå	56.0	0.86	0.0077	0.96
Sveg	-85.7	0.76	0.072	0.61
Falun	170	0.55	-0.051	0.72
Stockholm	-381	0.26	0.24	0.17
Malmslätt	-344	0.37	0.21	0.27
Västervik	-196	0.64	0.14	0.50
Växjö	-237	0.55	0.16	0.44

$ffmc_y = \beta_0 + \beta_1 y$

	Intercept		year (y)	
	β_0	*p*-value	β_1	*p*-value
Jokkmokk	80.8	<0.01	0.0038	0.73
Storuman	-5.11	0.80	0.047	<0.01
Umeå	96.7	<0.01	-0.0038	0.69
Sveg	53.6	0.02	0.018	0.11
Falun	24.7	0.16	0.033	<0.01
Stockholm	51.3	0.01	0.020	0.06
Malmslätt	67.2	<0.01	0.011	0.26
Västervik	10.1	0.58	0.040	<0.01
Växjö	80.4	<0.01	0.0047	0.63

$dmc_y = \beta_0 + \beta_1 y$

	Intercept		year (y)	
	β_0	*p*-value	β_1	*p*-value
Jokkmokk	-251	0.25	-0.10	0.34
Storuman	357	0.09	0.20	0.05
Umeå	150	0.58	-0.048	0.73
Sveg	-61.8	0.80	0.054	0.66
Falun	-29.6	0.90	0.042	0.72
Stockholm	-164	0.57	0.12	0.42
Malmslätt	-232	0.46	0.15	0.35
Västervik	-294	0.46	0.17	0.36
Växjö	-286	0.40	0.17	0.31

$dc_y = \beta_0 + \beta_1 y$

	Intercept		year (y)	
	β_0	*p*-value	β_1	*p*-value
Jokkmokk	2075	0.07	-0.94	0.10
Storuman	-547	0.59	0.39	0.46
Umeå	-773	0.58	0.53	0.45
Sveg	-385	0.75	0.30	0.63
Falun	2310	0.10	-1.03	0.15
Stockholm	-2343	0.11	1.34	0.07
Malmslätt	-1781	0.26	1.04	0.19
Västervik	578	0.72	-0.13	0.87
Växjö	-238	0.88	0.25	0.76

$t_y = \beta_0 + \beta_1 y$

	Intercept		year (y)	
	β_0	*p*-value	β_1	*p*-value
Jokkmokk	-17.0	0.74	0.018	0.48
Storuman	-146	<0.01	0.083	<0.01
Umeå	5.66	0.90	0.0075	0.73
Sveg	-92.1	0.07	0.057	0.02
Falun	-14.9	0.73	0.019	0.38
Stockholm	-84.0	0.07	0.054	0.02
Malmslätt	-78.5	0.07	0.051	0.02
Västervik	93.4	0.02	0.06	<0.01
Växjö	24.5	0.56	-0.0011	0.96

$t_{av,y} = \beta_0 + \beta_1 y$

	Intercept		year (y)	
	β_0	*p*-value	β_1	*p*-value
Jokkmokk	-19.1	0.58	0.017	0.34
Storuman	-58.8	0.06	0.037	0.02
Umeå	-20.6	0.59	0.018	0.36
Sveg	-12.6	0.75	0.014	0.49
Falun	47.3	0.26	-0.016	0.46
Stockholm	-124	<0.01	0.070	<0.01
Malmslätt	-111	0.02	0.064	<0.01
Västervik	-52.8	0.27	0.035	0.15
Växjö	-5.22	0.91	0.011	0.66

$rh_y = \beta_0 + \beta_1 y$

	Intercept		year (y)	
	β_0	*p*-value	β_1	*p*-value
Jokkmokk	-22.6	0.74	0.033	0.33
Storuman	361	<0.01	-0.16	<0.01
Umeå	6.28	0.93	0.0187	0.62
Sveg	153	0.021	-0.0577	0.08
Falun	305	<0.01	-0.134	<0.01
Stockholm	73.1	0.34	-0.0184	0.63
Malmslätt	45.0	0.6	-0.0016	0.97
Västervik	282	0.0004	-0.12	<0.01
Växjö	20.5	0.75	0.01	0.76

$vp_{wy} = \beta_0 + \beta_1 y$

	Intercept		year (y)	
	β_0	*p*-value	β_1	*p*-value
Jokkmokk	-0.37	0.36	0.00026	0.21
Storuman	-0.22	0.55	0.00018	0.32
Umeå	-0.24	0.60	0.00020	0.39
Sveg	-0.29	0.52	0.00022	0.34
Falun	0.91	0.06	-0.00038	0.11
Stockholm	-0.57	0.21	0.00037	0.11
Malmslätt	-0.84	0.08	0.00050	0.04
Västervik	0.027	0.95	0.00007	0.73
Växjö	0.15	0.75	0	0.97

$w_y = \beta_0 + \beta_1 y$

	Intercept		year (y)	
	β_0	*p*-value	β_1	*p*-value
Jokkmokk	2.23	0.92	0.0010	0.92
Storuman	40.0	0.01	-0.018	0.02
Umeå	-5.93	0.63	0.0054	0.38
Sveg	47.3	<0.01	-0.021	<0.01
Falun	-16.4	0.095	0.0098	0.049
Stockholm	32.1	<0.01	-0.014	<0.01
Malmslätt	11.7	0.32	-0.0038	0.52
Västervik	12.0	0.35	-0.0041	0.53
Växjö	-35.5	<0.01	0.020	<0.01

$p_{dmc,y} = \beta_0 + \beta_1 y$

	Intercept		year (y)	
	β_0	*p*-value	β_1	*p*-value
Jokkmokk	-166	0.06	0.088	0.05
Storuman	-91.7	0.29	0.0505	0.24
Umeå	23.1	0.68	-0.008	0.78
Sveg	-71.1	0.49	0.0417	0.42
Falun	-51.4	0.51	0.0311	0.43
Stockholm	-46.2	0.41	0.0267	0.34
Malmslätt	-22.1	0.74	0.0143	0.67
Västervik	-29.4	0.56	0.0181	0.48
Växjö	108	0.12	-0.0509	0.15

$$p_{dc,y} = \beta_0 + \beta_1 y$$

	Intercept		year (y)	
	β_0	*p*-value	β_1	*p*-value
Jokkmokk	-706	<0.01	0.333	<0.01
Storuman	-25	0.92	-0.0145	0.90
Umeå	21.1	0.92	-0.0445	0.69
Sveg	-43.4	0.87	-0.0005	0.99
Falun	-464	0.05	0.203	0.09
Stockholm	-136	0.47	0.0297	0.75
Malmslätt	-417	0.07	0.175	0.13
Västervik	-258	0.24	0.0929	0.40
Växjö	-26.7	0.9	-0.0188	0.87

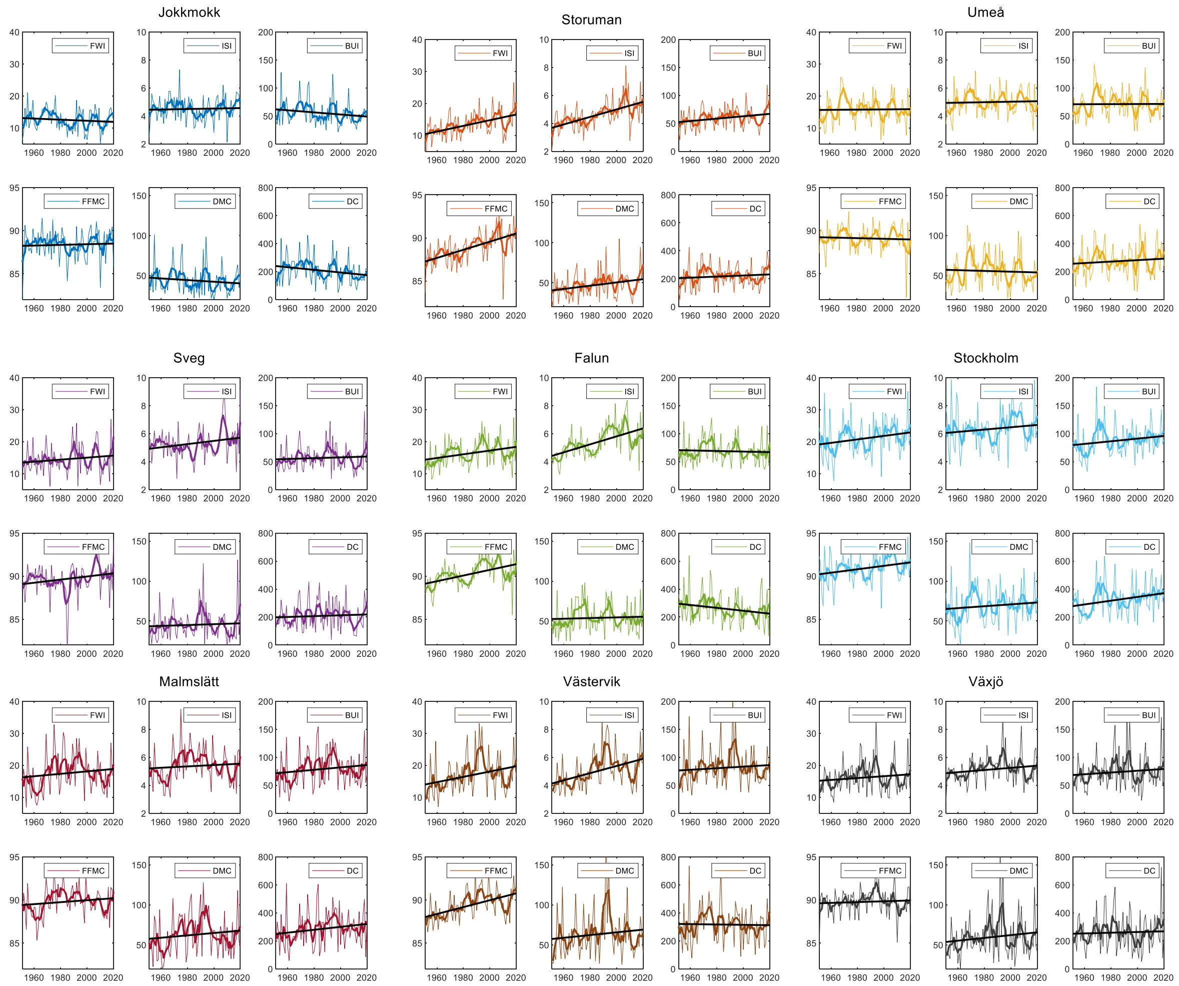

Supplementary Figure S3. The 7-day average of the indices and moisture codes concurrent with FWI-7x (the maximum 7-day running average of the FWI for each season) for each site and year. The black lines are linear regression to the data.
