# Supplementary material for: 70 Years of observational weather data show increasing fire danger for boreal Europe and reveal bias of ERA5 reanalysed data
Source: Sci Rep. 2025 Jun 20;15:20111. doi: 10.1038/s41598-025-04200-3 (PMC12181286; doi:10.1038/s41598-025-04200-3)
Supplement: Supplementary file 2 — Supplementary Material 2 [file 41598_2025_4200_MOESM2_ESM.pdf]

Appendix

Table A 1: Relative contributions to the 70-year linear change in FWI-7x for each site from the subindices, the moisture codes and the weather parameters. The categories "Residual" includes differences not captured by the linear trend model.

Site	FWI-7x linear change	Index	Contr. (%)	Moisture code	Contr. (%)	Weather param.	Contr. (%)
Jokkmokk	-1.2	ISI	+22 %	FFMC	+23 %	T	+98 %
		BUI	-130 %	DMC	-65 %	vp	+81 %
		Res	+8.5%	DC	-59 %	W	+3 %
				Res	+1 %	Prec	-136 %
						Res	+17 %
Storuman	+6.1	ISI	+68 %	FFMC	+67 %	T	+118 %
		BUI	+31 %	DMC	+25 %	vp	-17 %
		Res	+1 %	DC	+5 %	W	-8 %
				Res	+3 %	Prec	-8 %
						Res	+14 %
Umeå	+0.2	ISI	122 %	FFMC	-150 %	T	+368 %
		BUI	26 %	DMC	-142 %	vp	-446 %
		Res	-48 %	DC	+160 %	W	+91 %
				Res	+231 %	Prec	+120 %
						Res	-34 %
Sveg	+2.2	ISI	+77 %	FFMC	+65 %	T	+188 %
		BUI	+30 %	DMC	+20 %	vp	-50 %
		Res	-7 %	DC	+11 %	W	-30 %
				Res	+5 %	Prec	-22 %
						Res	-13 %
Falun	+4.0	ISI	+115 %	FFMC	+94 %	T	+37 %
		BUI	-11 %	DMC	+8 %	vp	+54 %
		Res	-4 %	DC	-19 %	W	+12 %
				Res	+17 %	Prec	-25 %
						Res	+22 %
Stockholm	+3.8	ISI	+38 %	FFMC	+71 %	T	+190 %
		BUI	+48 %	DMC	+20 %	vp	+68 %
		Res	+14 %	DC	+27 %	W	-29 %
				Res	-18 %	Prec	+18 %
						Res	+25 %
Malmslätt	+2.6	ISI	+32 %	FFMC	+53 %	T	+260 %
		BUI	+65 %	DMC	+38 %	vp	-127 %
		Res	+3 %	DC	+31 %	W	-9 %
				Res	-22 %	Prec	-26 %
						Res	+1 %
Västervik	+5.7	ISI	+80 %	FFMC	+89 %	T	+126 %

		BUI	+16 %	DMC	+17 %	vp	-10 %
		Res	+4 %	DC	-1 %	W	-4 %
				Res	-5 %	Prec	-11 %
						Res	-2 %
Växjö	+2.0	ISI	+60 %	FFMC	+22 %	T	+3 %
		BUI	+59 %	DMC	+55 %	vp	-2 %
		Res	-19 %	DC	+9 %	W	+43 %
				Res	+14 %	Prec	+47 %
						Res	+9 %
**Average**	+2.9	ISI	+75 %	FFMC	+74 %	T	+147 %
		BUI	+25 %	DMC	+20 %	vp	-34 %
		Res	±0 %	DC	+5 %	W	-4 %
				Res	+1 %	Prec	-18 %
						Res	+10 %
